# Supplementary material for: Structural Changes of the Trinuclear Copper Center in Bilirubin Oxidase upon Reduction
Source: Molecules. 2018 Dec 26;24(1):76. doi: 10.3390/molecules24010076 (PMC6337666; doi:10.3390/molecules24010076)
Supplement: Supplementary file 1 [file molecules-24-00076-s001.pdf]

Supplementary Materials

Structural Changes of the Trinuclear Copper Center in Bilirubin Oxidase upon Reduction

Takaki Tokiwa, Mitsuo Shoji, Vladimir Sladek, Naoki Shibata, Yoshiki Higuchi, Kunishige Kataoka, Takeshi Sakurai, Yasuteru Shigeta, and Fuminori Misaizu

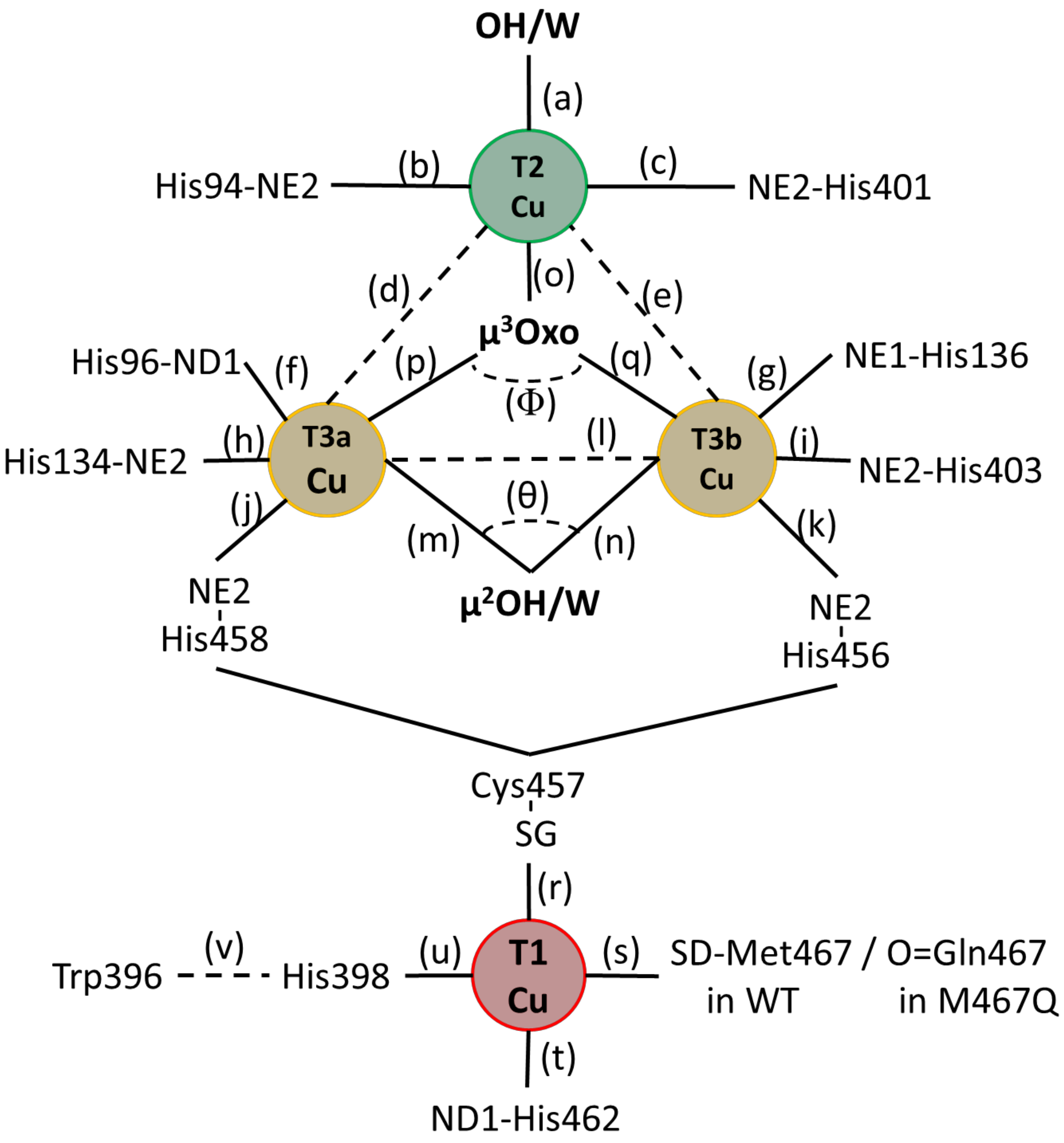

Figure S1. Schematic representation of the QM region in WT and M467Q BODs. WT BOD includes Met467 and covalent bond between Trp396 and His398. M467Q BOD includes Gln467 instead of Met467 and is lost its covalent bond.

Table S1.1 β molecular orbital energies for the Cu d orbitals in WT BOD. These orbitals are depicted in Figure 5 (WT).

States	main component	#MO	orbital energy/ eV
WT, NI	T1Cu	β HOMO	0.873
	μ^3-oxo	β LUMO	1.911
	T3Cu	β LUMO+2	2.187
	T3Cu	β LUMO+4	2.986
	T2Cu	β LUMO+5	3.592
WT, NI^{H+}	μ^3-oxo	β HOMO	-0.968
	T1Cu	β LUMO	-0.726
	T3Cu	β LUMO+1	0.661
	T2Cu	β LUMO+3	1.389
	T3Cu	β LUMO+4	1.646
WT, RO_0	T1Cu	β LUMO	-2.981
	T3Cu	β LUMO+1	-1.766
	T3Cu	β LUMO+2	-1.461
	T2Cu	β LUMO+3	-1.049
WT, FR_W	T3Cu	β HOMO-4	1.059
	T2Cu	β HOMO-3	1.044
	T3Cu	β HOMO-1	0.766
	T1Cu	β HOMO	0.721
WT, FR	T3Cu	β HOMO-5	1.032
	T2Cu	β HOMO-3	1.009
	T1Cu	β HOMO-1	0.653
	T3Cu	β HOMO	0.354

Table S1.2 β molecular orbital energies for the Cu d orbitals in M467Q BOD

States	main component	#MO	orbital energy/ eV
M467Q, NI	T1Cu	β HOMO	1.922
	μ^3-oxo	β LUMO	2.170
	T3Cu	β LUMO+2	2.804
	T3Cu	β LUMO+4	3.585
	T2Cu	β LUMO+5	4.048
M467Q, NI^{H+}	μ^3-oxo	β HOMO	-0.291
	T1Cu	β LUMO	-0.054
	T3Cu	β LUMO+2	1.683
	T2Cu	β LUMO+4	2.609
	T3Cu	β LUMO+5	2.786
M467Q, RO_0	T1Cu	β LUMO	-1.927
	T3Cu	β LUMO+1	-1.264
	T3Cu	β LUMO+2	-1.087
	T2Cu	β LUMO+3	-0.657
M467Q, FR_W	T2Cu	β HOMO-6	1.129
	T3Cu	β HOMO-3	1.411
	T3Cu	β HOMO-1	1.480
	T1Cu	β HOMO	2.099
M467Q, FR	T2Cu	β HOMO-5	0.989
	T3Cu	β HOMO-4	1.069
	T3Cu	β HOMO-2	1.432
	T1Cu	β HOMO	2.119

Table S2. Key atomic distances/Å, bond angles/degree around T1Cu and TNC. RMSDs/Å are calculated for the QM region between X-ray and QM/MM optimized structures.

	state	RMSD [Å]	(a) T2Cu-OH/W [Å]	(b) T2Cu-NE2(His94) [Å]	(c) T2Cu-NE2(His401) [Å]	(d) T2Cu-T3aCu [Å]	(e) T2Cu-T3bCu [Å]	(f) T3aCu-ND1(His96) [Å]	(g) T3bCu-NE2(His136) [Å]	(h) T3aCu-NE2(His134) [Å]	(i) T3bCu-NE2(His403) [Å]	(j) T3aCu-NE2(His458) [Å]	(k) T3bCu-NE2(His456) [Å]	(l) T3aCu-T3bCu [Å]	(m) T3aCu-μ2OH/W [Å]	(n) T3bCu-μ2OH/W [Å]	(o) T2Cu-μ3oxo [Å]	(p) T3aCu-μ3Oxo [Å]	(q) T3bCu-μ3Oxo [Å]	(r) T1Cu-SG(Cys457) [Å]	(s) T1Cu-SD(Met467) [Å]	(s) T1Cu-O(Gln467) [Å]	(t) T1Cu1-ND1(His462) [Å]	(u) T1Cu-ND1(His398) [Å]	(v) CD1(Trp396)-NE2(His398) [Å]	(*θ*) T3aCu-μ2OH/W-T3bCu [degree]	(*Φ*) T3aCu-μ3Oxo-T3bCu [degree]
WT BOD (PDB: 2XLL) [a]	-	-	3.14 (OH)	1.97	1.96	4.06	4.07	2.18	2.18	2.2	2.03	2.27	2.11	4.87	3.95 (μ2OH)	4.49 (μ2OH)	-	-	-	2.3	3.43(5)	-	2.20	1.95(5)	1.90	70.16	-
CueO Low X-ray dose (dataset 1, PDB: 3UAA) [b]	-	-	2.13 (OH)	2.04	1.97	3.65	3.61	2.07 (His103)	2.12 (His143)	2.34 (His144)	2.02 (His448)	2.19 (His501)	2.21 (His499)	3.22	2.07 (μ2OH)	1.82 (μ2OH)	1.83	2.08	2.26	-	-	-	-	-	-	112.04 (OH)	95.93
CueO High X-ray dose (dataset 6, PDB: 3UAE) [b]	-	-	2.34 (W)	1.96	1.92	4.4	3.98	1.91 (His103)	2.01 (His143)	1.94 (His144)	1.91 (His448)	2.12 (His501)	1.94 (His499)	4.96	2.77 (μ2W)	2.27 (μ2W)	-	-	-	-	-	-	-	-	-	159.44 (μ2W)	-
WT BOD (PDB: 6IQZ) [c]	-	-	3.11 (OH)	1.66	1.60	4.18	3.98	2.01	2.05	1,99	2.00	2.22	1.91	4.98	2.69 (μ2OH)	2.29 (μ2OH)	-	-	-	2.16	3.36	-	2.05	2.06	1.40	174.49	-
	NI	0.241475557	1.9 (OH)	1.92	1.92	3.84	3.75	2.16	2.10	2.32	2.04	2.07	2.14	3.14	1.94 (μ2OH)	1.9 (μ2OH)	2.07	2.02	2.18	2.2	3.46	-	1.98	2.20	1.39	109.3 (μ2OH)	96.26
	NIH+	0.240130462	2.11 (W)	1.9	1.95	3.60	3.56	2.16	2.19	2.40	2.05	2.06	2.16	3.06	1.95 (μ2OH)	1.9 (μ2OH)	1.94	1.99	2.07	2.2	3.33	-	2.00	2.06	1.40	105.39 (μ 2OH)	97.99
	ROW	0.233800696	2.12 (W)	1.88	1.90	3.52	3.59	2.18	2.11	2.08	2.03	2.12	2.22	3.38	2.58 (μ2OH)	1.99 (μ2OH)	2.03	1.94	2.11	2.17	3.25	-	1.98	2.01	1.40	94.69 (W)	113.26
WT BOD (QM/MM)	RO0	0.269486216	2.08 (W)	1.86	1.88	3.48	3.48	2.24	1.99	2.03	2.05	2.05	2.09	3.27	-	-	2.09	1.89	3	2.17	3.23	-	1.98	2.01	1.40	-	117.29
	RO1	0.236727161	1.85 (OH)	1.87	1.90	4.28	4.21	2.11	2.12	2.11	1.99	2.05	2.05	3.66	1.99 (μ2OH)	1.93 (μ2OH)	-	-	-	2.17	3.23	-	1.98	2.01	1.40	138.15	-
	FR	0.244951199	2.19 (W)	1.86	1.86	4.27	4.45	2.00	1.97	2.01	2.04	2.03	2.04	4.6	-	-	-	-	-	2.2	3.44	-	2.00	2.19	1.39	-	-
	FRw	0.220345187	2.19 (W)	1.86	1.86	4.34	4.52	2.04	1.97	2.03	1.99	2.09	2.2	4.79	2.45 (μ2W)	2.46 (μ2W)	-	-	-	2.22	3.31	-	2.02	2.10	1.39	155.23	-
Anaerobic M467Q BOD (PDB: 5ZD7) [c]	-	-	2.77 (OH)	1.87	1.79	4.20	4.02	1.95	2.02	2.00	1.95	2.17	2.04	4.78	2.58 (μ2OH)	2.22 (μ2OH)	-	-	-	2.25	-	2.72	1.99	2.01	2.88	170.39	-
	RO0	0.221005832	2.11 (W)	1.92	1.92	3.44	3.46	2.13	1.97	2.02	2.12	3.07	2.1	3.28	-	-	2.07	1.9	1.91	2.18	-	2.44	1.98	2.03	3.04	-	118.6
M467Q BOD (QM/MM)	FR	0.205966984	2.31 (W)	1.9	1.89	4.24	4.46	1.98	1.95	1.98	2.06	2.04	2.04	4.64	-	-	-	-	-	2.21	-	2.79	1.97	2.25	3.07	-	-
	FRw	0.216550936	2.33 (W)	1.9	1.89	4.16	4.48	2.00	2.00	2.03	2.03	2.09	2.05	4.67	2.34 (μ2W)	2.41 (μ2W)	-	-	-	2.21	-	2.77	1.98	2.24	3.07	158.34	-

[a] J. A. Cracknell, T. P. McNamara, E. D. Lowe, and C. F. Blanford, "rubin oxidase from Myrothecium verrucaria: X-ray determination of the complete crystal structure and a rational surface modification for enhanced electrocatalytic O2 reduction", Dalton Trans. 40, 6668 (2011).

[b] H. Komori, R. Sugiyama, K. Kataoka, Y. Higuchi, and T. Sakurai, "An O-Centered Structure of the Trinuclear Copper Center in the Cys500Ser/Glu506Gln Mutant of CueO and Structural Changes in Low to High X-Ray Dose Conditions", Angew. Chem. Int. Ed. 51(8), 1861–1864, (2012).

[c] M. Akter, T. Tokiwa, M. Shoji, K. Nishikawa, Y. Shigeta, T. Sakurai, Y. Higuchi, K. Kataoka, N. Shibata, "Redox potential-dependent formation of an unusual His–Trp bond in bilirubin oxidase", Chem. Eur. J., doi:10.1002/chem.201803798 (2018).

Table S3. Calculated Löwdin atomic spin densities. [a]

	state	T1Cu	T2Cu	T3aCu	T3bCu	μ3oxo	μ2OH/W	OH/W (connected T2Cu)	SG of Cys457	SD of Met467	OE1 of Gln467	CD2 of HID136	CE1 of HID136	CG of HID136
WT (QM/MM) CYS457	RO1	0.01	0.61	0.64	0.62	-	0.16 (μ2OH)	0.09 (W)	0.00	0.06	-	0.31	0.38	0.34
WT (QM/MM) CYX457	NI	0.00	0.59	0.66	0.64	1.07	0.28 (μ2OH)	0.2 (OH)	0.00	0.00	-	0.00	0.00	0.00
	NIH+ (W (connected	0.13	0.58	0.62	0.65	0.75	0.29 (μ2OH)	0.05 (W)	0.30	0.00	-	0.04	0.04	0.04
	NIH+ (μ2W)	0.17	0.59	0.58	0.40	0.91	0.09 (μ2W)	0.33 (OH)	0.38	0.00	-	0.00	0.00	0.00
	ROW	0.30	0.59	0.46	0.65	0.76	0.08 (W)	0.06 (W)	0.55	0.00	-	0.01	0.01	0.01
	RO0	0.31	0.62	0.65	0.53	0.63	-	0.06 (W)	0.57	0.00	-	0.10	0.10	0.11
	RO1	0.32	0.50	0.60	0.56	-	0.22 (μ2OH)	0.4 (OH)	0.57	0.00	-	0.10	0.10	0.11
	FR	0.00	0.00	0.00	0.00	-	-	0 (W)	0.00	0.00	-	0.00	0.00	0.00
	FRw	0.00	0.00	0.00	0.00	0.00	0 (μ2W)	0 (W)	0.00	0.00	-	0.00	0.00	0.00
M467Q BOD (QM/MM) CYS457	RO1	0.43	0.53	0.63	0.39	-	0.16 (μ2OH)	0.06 (W)	0.00	-	0.01	0.31	0.36	0.31
M467Q BOD (QM/MM) CYX457	RO0	0.33	0.62	0.64	0.59	0.60	-	0.06 (W)	0.57	-	0.00	0.01	0.00	0.01
	FR	0.00	0.00	0.00	0.00	-	-	0 (W)	0.00	-	0.00	0.00	0.00	0.00
	FRw	0.00	0.00	0.00	0.00	-	0 (μ2W)	0 (W)	0.00	-	0.00	0.00	0.00	0.00

[a] H_2O is named “W”.

211
WT NI

C	-0.791	-5.987	8.652
H	-0.061	-6.396	9.352
C	-0.612	-6.669	7.306
H	-1.350	-6.341	6.567
H	-0.758	-7.746	7.458
C	0.749	-6.403	6.750
N	1.899	-6.367	7.520
H	1.979	-6.791	8.440
C	2.953	-6.086	6.744
H	3.980	-6.079	7.070
N	2.530	-5.852	5.516
C	1.157	-6.025	5.509
H	0.540	-5.837	4.652
C	2.437	-2.784	5.344
H	2.991	-3.719	5.374
C	1.475	-2.816	4.142
H	0.942	-1.864	4.045
H	0.702	-3.566	4.350
C	2.099	-3.180	2.822
N	1.520	-4.077	1.919
C	2.316	-4.099	0.852
H	2.159	-4.704	-0.023
N	3.369	-3.270	1.019
H	4.174	-3.122	0.405
C	3.255	-2.692	2.260
H	4.023	-2.027	2.619
C	-4.854	-3.104	6.215
H	-5.778	-2.544	6.373
C	-4.558	-3.172	4.693
H	-4.341	-2.154	4.351
H	-5.465	-3.484	4.158
C	-3.422	-4.056	4.274
N	-3.504	-5.438	4.342
H	-4.305	-6.000	4.647
C	-2.334	-5.952	3.922
H	-2.119	-7.003	3.883
N	-1.488	-4.991	3.558
C	-2.161	-3.802	3.784
H	-1.698	-2.847	3.585
C	-4.742	-9.622	6.168
H	-4.866	-9.601	5.083
C	-3.255	-9.477	6.571
H	-3.052	-10.000	7.513
H	-3.028	-8.420	6.773
C	-2.256	-9.879	5.526
N	-2.287	-11.006	4.723
H	-3.018	-11.723	4.716
C	-1.221	-10.975	3.884
H	-1.051	-11.715	3.117
N	-0.486	-9.892	4.119
C	-1.131	-9.205	5.126
H	-0.753	-8.257	5.457
C	-2.967	-12.247	-15.391
H	-3.859	-11.624	-15.270
C	-2.982	-13.382	-14.334
H	-3.918	-13.921	-14.513
H	-3.088	-12.959	-13.330
C	-1.835	-14.358	-14.338
C	-0.759	-14.370	-13.475
N	0.102	-15.406	-13.773
H	0.968	-15.603	-13.278
C	-0.397	-16.100	-14.841
C	0.106	-17.229	-15.491
H	1.057	-17.667	-15.199
C	-0.678	-17.794	-16.491
H	-0.316	-18.666	-17.024
C	-1.937	-17.255	-16.822
H	-2.555	-17.772	-17.548
C	-2.417	-16.107	-16.201
H	-3.405	-15.722	-16.445
C	-1.638	-15.490	-15.206
C	0.651	-9.895	-10.193
H	-0.284	-9.895	-9.634
C	1.274	-11.310	-10.083
H	1.296	-11.596	-9.026
H	2.298	-11.330	-10.476
C	0.446	-12.318	-10.817
N	-0.759	-12.772	-10.309
C	-1.308	-13.526	-11.226
H	-2.249	-14.052	-11.139
N	-0.515	-13.586	-12.350
C	0.618	-12.821	-12.081
H	1.386	-12.681	-12.822
C	4.735	-7.947	-1.979
H	5.367	-7.083	-2.174
C	3.490	-7.545	-1.203
H	2.851	-8.405	-0.967
H	2.885	-6.889	-1.849
C	3.864	-6.901	0.072
N	4.865	-5.957	0.232
H	5.476	-5.599	-0.497
C	5.110	-5.796	1.552
H	5.811	-5.100	2.012
N	4.294	-6.582	2.227
C	3.525	-7.282	1.328
H	2.823	-8.045	1.605
C	7.121	-8.719	3.917
H	6.714	-7.704	3.867
C	6.272	-9.526	4.932
H	6.428	-9.130	5.942
H	6.578	-10.576	4.963
C	4.783	-9.449	4.701
N	3.947	-9.199	5.772
H	4.270	-9.125	6.741
C	2.672	-9.165	5.351
H	1.820	-8.993	5.991
N	2.624	-9.365	4.036
C	3.936	-9.559	3.621
H	4.176	-9.724	2.583
C	2.322	-12.922	-1.753
H	2.344	-13.919	-2.200
C	1.100	-12.789	-0.817
H	1.220	-13.536	-0.041
H	0.175	-13.057	-1.339
C	0.919	-11.464	-0.147
N	-0.060	-10.563	-0.503
H	-0.771	-10.690	-1.234
C	0.016	-9.498	0.314
H	-0.687	-8.685	0.283
N	1.022	-9.642	1.176
C	1.586	-10.872	0.899
H	2.402	-11.261	1.494
C	0.649	-11.371	-4.789
H	1.408	-10.701	-5.199
C	-0.131	-12.036	-5.906
H	-0.931	-12.671	-5.512
H	0.567	-12.672	-6.462
S	-0.834	-10.783	-7.054
C	-1.232	-8.198	-3.510
H	-1.596	-8.653	-2.585
C	-0.470	-6.896	-3.164
H	-0.610	-6.111	-3.917
H	0.613	-7.083	-3.149
C	-0.794	-6.396	-1.795
N	-2.052	-6.055	-1.328
H	-2.910	-6.075	-1.875
C	-1.950	-5.697	-0.023
H	-2.782	-5.402	0.598
N	-0.687	-5.781	0.373
C	0.031	-6.236	-0.714
H	1.081	-6.463	-0.626
C	-5.441	-13.891	-6.169
H	-5.870	-14.784	-6.626
C	-4.265	-13.365	-7.009
H	-3.472	-14.124	-7.079
H	-3.777	-12.503	-6.534
C	-4.634	-12.999	-8.409
N	-3.689	-12.431	-9.249
C	-4.278	-12.229	-10.416
H	-3.819	-11.755	-11.270
N	-5.564	-12.639	-10.370
H	-6.185	-12.671	-11.174
C	-5.806	-13.132	-9.104
H	-6.759	-13.546	-8.821
C	-1.376	-17.026	-4.089
H	-0.851	-17.937	-4.397
C	-1.489	-16.056	-5.277
H	-1.888	-15.085	-4.965
H	-2.221	-16.474	-5.963
C	-0.170	-15.844	-6.034
H	0.303	-16.804	-6.278
H	0.533	-15.279	-5.415
S	-0.327	-14.896	-7.616
C	-0.379	-16.270	-8.809
H	-0.528	-15.834	-9.803
H	-1.188	-16.974	-8.609
H	0.556	-16.831	-8.788
CU	-1.877	-11.965	-8.591
CU	0.930	-8.714	3.106
CU	0.238	-5.787	2.221
CU	3.618	-6.257	3.991
H	-1.318	-7.950	2.261
O	1.805	-6.713	3.106
H	5.562	-6.318	5.665
O	5.371	-6.230	4.712
O	-0.490	-7.537	2.634
O	-2.869	-8.631	2.112
H	-3.204	-8.824	3.003
H	-2.962	-9.520	1.657
O	6.548	-6.529	7.265
H	5.920	-6.894	7.954
H	6.811	-5.655	7.623
O	6.353	-4.061	3.651
H	6.125	-4.912	4.125
H	7.314	-3.954	3.745
H	-1.691	-6.057	8.982
H	-0.511	-4.851	8.529
H	1.968	-2.643	6.174
H	3.253	-1.932	5.217
H	-4.122	-2.730	6.721
H	-5.062	-4.187	6.619
H	-5.287	-8.943	6.579
H	-5.128	-10.690	6.491
H	-2.893	-12.577	-16.291
H	-2.042	-11.538	-15.212
H	0.526	-9.606	-11.105
H	1.364	-9.131	-9.646
H	4.486	-8.393	-2.779
H	5.387	-8.694	-1.340
H	7.161	-9.125	3.037
H	8.232	-8.611	4.333
H	3.158	-12.711	-1.318
H	2.157	-12.172	-2.636
H	1.040	-12.006	-4.178
H	-0.093	-10.686	-4.171
H	-0.685	-8.829	-3.993
H	-2.220	-7.943	-4.116
H	-6.127	-13.229	-6.004
H	-5.001	-14.247	-5.136
H	-2.239	-17.264	-3.715
H	-0.688	-16.525	-3.266

212

WT NIH+

C	-0.804	-5.992	8.647
H	-0.070	-6.411	9.337
C	-0.680	-6.694	7.307
H	-1.437	-6.362	6.587
H	-0.842	-7.766	7.483
C	0.657	-6.456	6.711
N	1.827	-6.401	7.448
H	1.939	-6.811	8.373
C	2.844	-6.091	6.640
H	3.871	-6.027	6.953
N	2.373	-5.882	5.419
C	1.013	-6.091	5.453
H	0.376	-5.974	4.601
C	2.432	-2.784	5.339
H	2.983	-3.720	5.362
C	1.462	-2.793	4.139
H	0.923	-1.843	4.069
H	0.697	-3.554	4.337
C	2.083	-3.127	2.810
N	1.550	-4.068	1.927
C	2.351	-4.086	0.867
H	2.236	-4.732	0.014
N	3.366	-3.206	1.010
H	4.161	-3.047	0.387
C	3.213	-2.593	2.234
H	3.910	-1.840	2.564
C	-4.874	-3.093	6.240
H	-5.795	-2.538	6.433
C	-4.621	-3.130	4.710
H	-4.420	-2.104	4.381
H	-5.539	-3.439	4.193
C	-3.488	-3.999	4.258
N	-3.569	-5.379	4.311
H	-4.366	-5.946	4.617
C	-2.394	-5.886	3.892
H	-2.174	-6.935	3.850
N	-1.546	-4.922	3.542
C	-2.225	-3.738	3.777
H	-1.764	-2.780	3.590
C	-4.748	-9.625	6.160
H	-4.884	-9.605	5.078
C	-3.258	-9.476	6.551
H	-3.044	-9.992	7.495
H	-3.026	-8.419	6.746
C	-2.272	-9.896	5.511
N	-2.301	-11.027	4.728
H	-3.037	-11.742	4.726
C	-1.230	-11.001	3.888
H	-1.054	-11.750	3.132
N	-0.487	-9.914	4.109
C	-1.134	-9.220	5.092
H	-0.763	-8.268	5.422
C	-2.967	-12.246	-15.392
H	-3.857	-11.620	-15.272
C	-2.982	-13.377	-14.333
H	-3.916	-13.922	-14.512
H	-3.094	-12.953	-13.330
C	-1.834	-14.355	-14.334
C	-0.761	-14.376	-13.471
N	0.102	-15.409	-13.764
H	0.970	-15.608	-13.274
C	-0.398	-16.100	-14.836
C	0.104	-17.233	-15.478
H	1.053	-17.672	-15.182
C	-0.678	-17.797	-16.479
H	-0.316	-18.669	-17.012
C	-1.935	-17.254	-16.816
H	-2.550	-17.773	-17.543
C	-2.416	-16.104	-16.199
H	-3.403	-15.721	-16.446
C	-1.636	-15.489	-15.203
C	0.648	-9.893	-10.191
H	-0.289	-9.879	-9.638
C	1.271	-11.308	-10.084
H	1.317	-11.603	-9.030
H	2.289	-11.326	-10.489
C	0.438	-12.314	-10.815
N	-0.778	-12.755	-10.320
C	-1.323	-13.524	-11.235
H	-2.267	-14.042	-11.146
N	-0.519	-13.594	-12.338
C	0.612	-12.828	-12.071
H	1.389	-12.704	-12.806
C	4.743	-7.944	-1.975
H	5.379	-7.083	-2.174
C	3.522	-7.533	-1.161
H	2.879	-8.389	-0.922
H	2.908	-6.856	-1.771
C	3.947	-6.932	0.123
N	4.908	-5.940	0.280
H	5.487	-5.547	-0.457
C	5.201	-5.834	1.590
H	5.951	-5.179	2.006
N	4.460	-6.686	2.284
C	3.695	-7.389	1.370
H	3.002	-8.159	1.636
C	7.123	-8.718	3.923
H	6.728	-7.701	3.856
C	6.283	-9.536	4.938
H	6.455	-9.170	5.957
H	6.590	-10.585	4.938
C	4.795	-9.445	4.723
N	3.965	-9.163	5.792
H	4.279	-9.121	6.765
C	2.689	-9.134	5.371
H	1.841	-8.937	6.009
N	2.638	-9.353	4.062
C	3.944	-9.566	3.650
H	4.179	-9.760	2.617
C	2.309	-12.914	-1.736
H	2.322	-13.915	-2.177
C	1.092	-12.765	-0.797
H	1.216	-13.504	-0.011
H	0.161	-13.040	-1.304
C	0.921	-11.436	-0.136
N	-0.091	-10.553	-0.444
H	-0.836	-10.685	-1.137
C	-0.002	-9.496	0.386
H	-0.729	-8.703	0.409
N	1.038	-9.630	1.207
C	1.619	-10.840	0.888
H	2.471	-11.217	1.436
C	0.638	-11.379	-4.789
H	1.390	-10.714	-5.219
C	-0.164	-12.072	-5.873
H	-0.958	-12.702	-5.457
H	0.507	-12.712	-6.455
S	-0.927	-10.866	-7.044
C	-1.233	-8.200	-3.509
H	-1.600	-8.659	-2.588
C	-0.467	-6.905	-3.150
H	-0.591	-6.116	-3.901
H	0.615	-7.103	-3.127
C	-0.793	-6.408	-1.779
N	-2.048	-6.056	-1.314
H	-2.908	-6.071	-1.856
C	-1.938	-5.695	-0.010
H	-2.766	-5.385	0.611
N	-0.677	-5.785	0.385
C	0.037	-6.247	-0.701
H	1.083	-6.487	-0.612
C	-5.444	-13.895	-6.168
H	-5.874	-14.786	-6.624
C	-4.266	-13.372	-7.002
H	-3.478	-14.137	-7.074
H	-3.779	-12.512	-6.520
C	-4.628	-12.998	-8.402
N	-3.680	-12.424	-9.232
C	-4.263	-12.218	-10.406
H	-3.797	-11.737	-11.254
N	-5.543	-12.637	-10.371
H	-6.162	-12.665	-11.178
C	-5.793	-13.138	-9.108
H	-6.744	-13.565	-8.838
C	-1.375	-17.025	-4.088
H	-0.853	-17.937	-4.397
C	-1.492	-16.055	-5.275
H	-1.895	-15.085	-4.962
H	-2.222	-16.474	-5.962
C	-0.173	-15.842	-6.031
H	0.300	-16.799	-6.281
H	0.535	-15.281	-5.413
S	-0.322	-14.894	-7.618
C	-0.379	-16.267	-8.813
H	-0.529	-15.833	-9.808
H	-1.189	-16.966	-8.605
H	0.556	-16.827	-8.787
CU	-1.789	-12.088	-8.657
CU	0.980	-8.622	3.111
CU	0.267	-5.782	2.219
CU	3.439	-6.167	3.867
H	-1.306	-7.931	2.323
O	1.853	-6.758	2.915
O	5.065	-5.330	4.910
H	5.505	-5.845	5.635
H	5.802	-4.964	4.356
O	-0.503	-7.498	2.722
O	-2.851	-8.659	2.127
H	-3.224	-8.841	3.007
H	-2.951	-9.549	1.674
O	6.363	-6.422	7.104
H	5.797	-6.857	7.808
H	6.714	-5.627	7.562
O	7.099	-4.279	3.607
H	7.873	-4.415	4.202
H	7.208	-3.363	3.294
H	-1.697	-6.049	8.987
H	-0.511	-4.857	8.524
H	1.967	-2.644	6.171
H	3.249	-1.932	5.216
H	-4.130	-2.726	6.732
H	-5.070	-4.181	6.632
H	-5.288	-8.945	6.575
H	-5.130	-10.691	6.489
H	-2.895	-12.578	-16.291
H	-2.043	-11.536	-15.215
H	0.525	-9.606	-11.104
H	1.363	-9.129	-9.646
H	4.479	-8.384	-2.776
H	5.394	-8.698	-1.345
H	7.163	-9.124	3.042
H	8.234	-8.611	4.336
H	3.148	-12.704	-1.303
H	2.151	-12.169	-2.626
H	1.036	-12.006	-4.175
H	-0.096	-10.688	-4.169
H	-0.684	-8.832	-3.993
H	-2.221	-7.943	-4.116
H	-6.127	-13.231	-6.004
H	-5.005	-14.249	-5.135
H	-2.238	-17.263	-3.715
H	-0.687	-16.524	-3.265

213

WT ROW

C	-0.809	-5.986	8.668
H	-0.076	-6.399	9.362
C	-0.699	-6.714	7.347
H	-1.458	-6.394	6.623
H	-0.864	-7.782	7.541
C	0.628	-6.481	6.749
N	1.809	-6.406	7.462
H	1.944	-6.802	8.390
C	2.804	-6.077	6.635
H	3.835	-5.999	6.929
N	2.298	-5.869	5.427
C	0.946	-6.101	5.492
H	0.289	-5.977	4.660
C	2.440	-2.770	5.341
H	2.988	-3.708	5.356
C	1.497	-2.742	4.128
H	0.950	-1.794	4.078
H	0.736	-3.518	4.279
C	2.153	-3.020	2.804
N	1.589	-3.891	1.879
C	2.408	-3.922	0.835
H	2.273	-4.528	-0.046
N	3.467	-3.111	1.032
H	4.268	-2.948	0.414
C	3.327	-2.533	2.273
H	4.061	-1.831	2.632
C	-4.843	-3.109	6.202
H	-5.767	-2.545	6.341
C	-4.534	-3.184	4.684
H	-4.304	-2.171	4.338
H	-5.438	-3.491	4.141
C	-3.409	-4.083	4.262
N	-3.501	-5.461	4.358
H	-4.302	-6.003	4.700
C	-2.367	-6.006	3.894
H	-2.170	-7.058	3.891
N	-1.528	-5.059	3.473
C	-2.170	-3.851	3.711
H	-1.701	-2.909	3.477
C	-4.735	-9.625	6.172
H	-4.857	-9.604	5.086
C	-3.250	-9.485	6.592
H	-3.063	-9.995	7.543
H	-3.020	-8.427	6.782
C	-2.240	-9.914	5.570
N	-2.263	-11.054	4.787
H	-2.991	-11.778	4.803
C	-1.199	-11.034	3.947
H	-1.012	-11.792	3.203
N	-0.465	-9.940	4.160
C	-1.112	-9.241	5.156
H	-0.726	-8.296	5.489
C	-2.966	-12.247	-15.390
H	-3.859	-11.626	-15.272
C	-2.978	-13.381	-14.334
H	-3.913	-13.923	-14.514
H	-3.089	-12.956	-13.330
C	-1.831	-14.361	-14.335
C	-0.756	-14.388	-13.477
N	0.101	-15.425	-13.771
H	0.978	-15.619	-13.295
C	-0.405	-16.111	-14.844
C	0.094	-17.245	-15.491
H	1.044	-17.685	-15.203
C	-0.692	-17.799	-16.494
H	-0.327	-18.664	-17.038
C	-1.950	-17.254	-16.824
H	-2.570	-17.766	-17.549
C	-2.423	-16.103	-16.205
H	-3.410	-15.714	-16.446
C	-1.640	-15.497	-15.205
C	0.649	-9.893	-10.191
H	-0.290	-9.876	-9.642
C	1.276	-11.308	-10.093
H	1.348	-11.615	-9.043
H	2.290	-11.321	-10.506
C	0.437	-12.314	-10.821
N	-0.787	-12.746	-10.337
C	-1.326	-13.527	-11.252
H	-2.268	-14.047	-11.171
N	-0.510	-13.607	-12.339
C	0.621	-12.847	-12.068
H	1.403	-12.731	-12.798
C	4.743	-7.947	-1.982
H	5.376	-7.084	-2.182
C	3.503	-7.539	-1.204
H	2.857	-8.394	-0.969
H	2.900	-6.872	-1.842
C	3.878	-6.905	0.076
N	4.910	-5.999	0.266
H	5.552	-5.659	-0.445
C	5.089	-5.815	1.584
H	5.859	-5.201	2.027
N	4.202	-6.548	2.243
C	3.465	-7.247	1.313
H	2.693	-7.943	1.567
C	7.117	-8.709	3.917
H	6.737	-7.686	3.844
C	6.249	-9.505	4.931
H	6.417	-9.131	5.948
H	6.534	-10.561	4.947
C	4.759	-9.386	4.701
N	3.923	-9.163	5.783
H	4.237	-9.154	6.759
C	2.650	-9.092	5.360
H	1.801	-8.929	6.007
N	2.601	-9.232	4.038
C	3.912	-9.435	3.616
H	4.148	-9.584	2.574
C	2.346	-12.944	-1.774
H	2.390	-13.932	-2.239
C	1.136	-12.860	-0.823
H	1.302	-13.605	-0.057
H	0.206	-13.158	-1.318
C	0.936	-11.546	-0.144
N	-0.050	-10.676	-0.535
H	-0.748	-10.826	-1.274
C	0.005	-9.584	0.240
H	-0.723	-8.801	0.140
N	0.996	-9.680	1.125
C	1.585	-10.909	0.889
H	2.403	-11.258	1.503
C	0.622	-11.381	-4.792
H	1.375	-10.727	-5.236
C	-0.199	-12.097	-5.841
H	-1.006	-12.696	-5.406
H	0.445	-12.768	-6.418
S	-0.959	-10.944	-7.057
C	-1.234	-8.200	-3.501
H	-1.607	-8.667	-2.586
C	-0.471	-6.901	-3.126
H	-0.593	-6.110	-3.875
H	0.610	-7.100	-3.096
C	-0.810	-6.398	-1.755
N	-2.078	-6.054	-1.316
H	-2.925	-6.068	-1.881
C	-2.004	-5.687	-0.015
H	-2.852	-5.382	0.580
N	-0.748	-5.770	0.417
C	-0.002	-6.223	-0.659
H	1.053	-6.419	-0.551
C	-5.447	-13.899	-6.168
H	-5.881	-14.791	-6.620
C	-4.263	-13.387	-7.003
H	-3.482	-14.160	-7.067
H	-3.777	-12.529	-6.517
C	-4.607	-13.008	-8.407
N	-3.640	-12.449	-9.229
C	-4.203	-12.230	-10.414
H	-3.721	-11.756	-11.255
N	-5.487	-12.627	-10.389
H	-6.103	-12.637	-11.203
C	-5.763	-13.125	-9.131
H	-6.726	-13.532	-8.873
C	-1.379	-17.020	-4.089
H	-0.857	-17.929	-4.404
C	-1.507	-16.040	-5.268
H	-1.905	-15.072	-4.941
H	-2.247	-16.450	-5.951
C	-0.195	-15.826	-6.035
H	0.272	-16.782	-6.295
H	0.521	-15.268	-5.425
S	-0.356	-14.883	-7.628
C	-0.383	-16.255	-8.826
H	-0.520	-15.826	-9.824
H	-1.191	-16.958	-8.626
H	0.556	-16.806	-8.780
CU	-1.760	-12.151	-8.679
CU	0.876	-8.687	3.110
CU	0.153	-5.478	2.317
CU	3.265	-6.107	3.833
O	1.534	-6.686	2.936
H	-1.705	-8.416	2.492
H	-0.770	-7.529	1.537
O	4.949	-5.298	4.829
H	5.376	-5.788	5.580
H	5.704	-4.922	4.300
O	-0.807	-7.872	2.442
O	-3.053	-8.883	2.463
H	-3.458	-9.006	3.339
H	-3.129	-9.813	2.019
O	6.244	-6.350	7.054
H	5.732	-6.813	7.784
H	6.643	-5.582	7.517
O	7.067	-4.281	3.633
H	7.829	-4.450	4.232
H	7.234	-3.378	3.306
H	-1.701	-6.035	9.001
H	-0.514	-4.853	8.530
H	1.969	-2.640	6.172
H	3.257	-1.923	5.230
H	-4.116	-2.730	6.712
H	-5.058	-4.187	6.615
H	-5.281	-8.944	6.579
H	-5.125	-10.691	6.495
H	-2.892	-12.579	-16.290
H	-2.041	-11.537	-15.213
H	0.524	-9.604	-11.103
H	1.363	-9.129	-9.646
H	4.489	-8.395	-2.781
H	5.391	-8.696	-1.343
H	7.156	-9.119	3.039
H	8.230	-8.611	4.335
H	3.176	-12.723	-1.335
H	2.168	-12.181	-2.647
H	1.025	-12.003	-4.177
H	-0.102	-10.685	-4.167
H	-0.685	-8.829	-3.988
H	-2.222	-7.941	-4.113
H	-6.128	-13.233	-6.006
H	-5.008	-14.251	-5.134
H	-2.240	-17.260	-3.714
H	-0.688	-16.523	-3.265

210
WT RO0

C	-0.807	-5.981	8.677
H	-0.075	-6.389	9.373
C	-0.692	-6.713	7.362
H	-1.455	-6.405	6.636
H	-0.841	-7.783	7.557
C	0.629	-6.465	6.758
N	1.825	-6.423	7.445
H	1.974	-6.827	8.369
C	2.805	-6.085	6.607
H	3.844	-6.027	6.880
N	2.277	-5.841	5.414
C	0.921	-6.046	5.508
H	0.237	-5.862	4.710
C	2.441	-2.772	5.342
H	2.990	-3.711	5.360
C	1.492	-2.752	4.133
H	0.962	-1.796	4.076
H	0.721	-3.514	4.300
C	2.138	-3.059	2.810
N	1.580	-3.963	1.911
C	2.380	-3.983	0.849
H	2.242	-4.600	-0.023
N	3.422	-3.141	1.016
H	4.213	-2.967	0.385
C	3.288	-2.549	2.251
H	4.004	-1.817	2.591
C	-4.823	-3.123	6.177
H	-5.746	-2.554	6.286
C	-4.489	-3.236	4.667
H	-4.249	-2.232	4.303
H	-5.392	-3.541	4.120
C	-3.375	-4.154	4.237
N	-3.483	-5.539	4.276
H	-4.273	-6.080	4.651
C	-2.387	-6.085	3.721
H	-2.233	-7.150	3.597
N	-1.555	-5.119	3.315
C	-2.162	-3.913	3.639
H	-1.695	-2.968	3.417
C	-4.732	-9.624	6.175
H	-4.843	-9.602	5.090
C	-3.244	-9.484	6.594
H	-3.042	-10.040	7.517
H	-3.020	-8.436	6.834
C	-2.246	-9.858	5.540
N	-2.284	-10.961	4.709
H	-3.007	-11.689	4.712
C	-1.252	-10.904	3.840
H	-1.097	-11.627	3.056
N	-0.511	-9.821	4.086
C	-1.130	-9.165	5.137
H	-0.732	-8.241	5.510
C	-2.968	-12.245	-15.392
H	-3.860	-11.623	-15.274
C	-2.982	-13.377	-14.333
H	-3.917	-13.918	-14.509
H	-3.090	-12.950	-13.329
C	-1.837	-14.358	-14.335
C	-0.760	-14.389	-13.479
N	0.092	-15.430	-13.770
H	0.970	-15.627	-13.297
C	-0.408	-16.103	-14.855
C	0.085	-17.238	-15.504
H	1.026	-17.692	-15.205
C	-0.714	-17.802	-16.489
H	-0.359	-18.672	-17.029
C	-1.973	-17.254	-16.814
H	-2.593	-17.762	-17.542
C	-2.438	-16.098	-16.200
H	-3.425	-15.709	-16.439
C	-1.646	-15.491	-15.208
C	0.648	-9.892	-10.190
H	-0.292	-9.875	-9.641
C	1.273	-11.308	-10.093
H	1.341	-11.615	-9.044
H	2.287	-11.320	-10.506
C	0.436	-12.314	-10.822
N	-0.787	-12.749	-10.339
C	-1.325	-13.531	-11.253
H	-2.266	-14.053	-11.169
N	-0.511	-13.609	-12.340
C	0.617	-12.843	-12.072
H	1.400	-12.729	-12.800
C	4.756	-7.953	-1.996
H	5.398	-7.098	-2.198
C	3.511	-7.529	-1.246
H	2.858	-8.380	-1.010
H	2.921	-6.870	-1.903
C	3.863	-6.887	0.030
N	4.911	-6.010	0.258
H	5.580	-5.672	-0.430
C	5.031	-5.818	1.582
H	5.795	-5.217	2.054
N	4.088	-6.516	2.198
C	3.376	-7.201	1.247
H	2.563	-7.857	1.478
C	7.125	-8.703	3.916
H	6.744	-7.681	3.842
C	6.267	-9.492	4.935
H	6.449	-9.122	5.951
H	6.542	-10.550	4.947
C	4.780	-9.363	4.728
N	3.967	-9.193	5.829
H	4.304	-9.192	6.793
C	2.684	-9.131	5.450
H	1.851	-9.011	6.127
N	2.608	-9.228	4.129
C	3.909	-9.378	3.661
H	4.122	-9.461	2.608
C	2.259	-12.862	-1.674
H	2.211	-13.879	-2.068
C	1.082	-12.630	-0.699
H	1.223	-13.328	0.126
H	0.136	-12.932	-1.163
C	0.885	-11.271	-0.084
N	-0.222	-10.488	-0.357
H	-0.997	-10.694	-0.993
C	-0.246	-9.439	0.484
H	-1.076	-8.752	0.557
N	0.821	-9.468	1.274
C	1.531	-10.610	0.934
H	2.421	-10.892	1.477
C	0.621	-11.386	-4.791
H	1.372	-10.734	-5.240
C	-0.201	-12.109	-5.835
H	-1.009	-12.705	-5.395
H	0.442	-12.785	-6.408
S	-0.967	-10.960	-7.052
C	-1.233	-8.210	-3.510
H	-1.602	-8.674	-2.590
C	-0.467	-6.913	-3.127
H	-0.610	-6.106	-3.855
H	0.615	-7.105	-3.121
C	-0.788	-6.461	-1.735
N	-2.047	-6.119	-1.264
H	-2.900	-6.054	-1.815
C	-1.951	-5.843	0.058
H	-2.780	-5.554	0.685
N	-0.695	-5.984	0.463
C	0.032	-6.378	-0.639
H	1.081	-6.606	-0.552
C	-5.446	-13.898	-6.167
H	-5.880	-14.790	-6.620
C	-4.261	-13.385	-7.000
H	-3.477	-14.156	-7.058
H	-3.778	-12.523	-6.516
C	-4.603	-13.011	-8.405
N	-3.635	-12.457	-9.228
C	-4.199	-12.233	-10.412
H	-3.716	-11.761	-11.253
N	-5.483	-12.629	-10.387
H	-6.101	-12.635	-11.200
C	-5.761	-13.125	-9.128
H	-6.724	-13.531	-8.870
C	-1.379	-17.020	-4.088
H	-0.858	-17.930	-4.405
C	-1.506	-16.041	-5.268
H	-1.904	-15.073	-4.943
H	-2.246	-16.452	-5.950
C	-0.194	-15.829	-6.035
H	0.271	-16.786	-6.297
H	0.525	-15.274	-5.425
S	-0.351	-14.884	-7.628
C	-0.380	-16.254	-8.827
H	-0.512	-15.824	-9.825
H	-1.191	-16.955	-8.629
H	0.558	-16.808	-8.779
CU	-1.757	-12.167	-8.676
CU	0.857	-8.679	3.208
CU	0.127	-5.620	2.302
CU	3.205	-6.179	3.820
H	5.672	-5.077	4.334
O	1.411	-6.833	2.978
H	5.404	-6.035	5.573
O	4.975	-5.629	4.774
O	-2.841	-8.733	2.282
H	-3.726	-8.573	2.648
H	-2.933	-9.676	1.939
O	6.249	-6.419	7.083
H	5.721	-6.864	7.820
H	6.630	-5.636	7.537
O	6.930	-4.134	3.885
H	7.723	-4.249	4.465
H	7.144	-3.359	3.337
H	-1.700	-6.031	9.007
H	-0.515	-4.849	8.536
H	1.971	-2.639	6.172
H	3.258	-1.925	5.227
H	-4.104	-2.737	6.695
H	-5.048	-4.195	6.602
H	-5.278	-8.942	6.580
H	-5.125	-10.690	6.495
H	-2.894	-12.578	-16.290
H	-2.046	-11.532	-15.217
H	0.524	-9.604	-11.102
H	1.363	-9.128	-9.646
H	4.503	-8.405	-2.788
H	5.397	-8.699	-1.347
H	7.161	-9.117	3.039
H	8.235	-8.606	4.333
H	3.121	-12.682	-1.274
H	2.116	-12.144	-2.596
H	1.024	-12.004	-4.171
H	-0.105	-10.689	-4.173
H	-0.688	-8.841	-4.003
H	-2.222	-7.947	-4.116
H	-6.127	-13.232	-6.005
H	-5.007	-14.251	-5.133
H	-2.240	-17.260	-3.714
H	-0.689	-16.522	-3.266

210

WT RO1

C	-0.789	-5.992	8.637
H	-0.062	-6.407	9.337
C	-0.612	-6.672	7.282
H	-1.348	-6.330	6.547
H	-0.775	-7.745	7.444
C	0.759	-6.442	6.717
N	1.882	-6.340	7.522
H	1.932	-6.705	8.471
C	2.962	-6.112	6.771
H	3.973	-6.041	7.134
N	2.585	-5.993	5.510
C	1.216	-6.193	5.454
H	0.656	-6.162	4.537
C	2.440	-2.781	5.349
H	2.992	-3.718	5.386
C	1.475	-2.805	4.154
H	0.936	-1.855	4.072
H	0.710	-3.566	4.362
C	2.094	-3.147	2.831
N	1.488	-4.018	1.921
C	2.265	-4.021	0.833
H	2.091	-4.599	-0.058
N	3.331	-3.215	1.006
H	4.125	-3.056	0.378
C	3.247	-2.662	2.263
H	4.027	-2.010	2.621
C	-4.872	-3.095	6.240
H	-5.791	-2.537	6.422
C	-4.610	-3.149	4.713
H	-4.405	-2.130	4.371
H	-5.523	-3.466	4.191
C	-3.476	-4.029	4.282
N	-3.552	-5.409	4.358
H	-4.349	-5.964	4.691
C	-2.384	-5.931	3.951
H	-2.184	-6.988	3.926
N	-1.539	-4.962	3.588
C	-2.215	-3.771	3.800
H	-1.755	-2.818	3.592
C	-4.737	-9.625	6.185
H	-4.831	-9.596	5.097
C	-3.263	-9.476	6.634
H	-3.088	-9.979	7.595
H	-3.021	-8.418	6.812
C	-2.279	-9.939	5.625
N	-2.351	-11.071	4.863
H	-3.102	-11.773	4.908
C	-1.317	-11.075	3.982
H	-1.185	-11.837	3.230
N	-0.542	-9.997	4.171
C	-1.130	-9.285	5.168
H	-0.720	-8.345	5.485
C	-2.967	-12.245	-15.392
H	-3.857	-11.619	-15.273
C	-2.981	-13.376	-14.333
H	-3.916	-13.919	-14.510
H	-3.090	-12.951	-13.330
C	-1.837	-14.359	-14.336
C	-0.761	-14.389	-13.478
N	0.094	-15.428	-13.772
H	0.971	-15.627	-13.297
C	-0.413	-16.110	-14.848
C	0.079	-17.246	-15.494
H	1.028	-17.691	-15.206
C	-0.711	-17.799	-16.493
H	-0.358	-18.672	-17.029
C	-1.966	-17.246	-16.824
H	-2.592	-17.763	-17.543
C	-2.436	-16.096	-16.203
H	-3.423	-15.708	-16.440
C	-1.643	-15.488	-15.212
C	0.648	-9.892	-10.190
H	-0.292	-9.875	-9.640
C	1.273	-11.308	-10.093
H	1.341	-11.616	-9.044
H	2.287	-11.321	-10.505
C	0.436	-12.313	-10.823
N	-0.787	-12.749	-10.338
C	-1.324	-13.532	-11.253
H	-2.266	-14.054	-11.169
N	-0.511	-13.610	-12.340
C	0.617	-12.844	-12.072
H	1.400	-12.729	-12.800
C	4.713	-7.946	-1.958
H	5.333	-7.073	-2.154
C	3.468	-7.575	-1.156
H	2.862	-8.455	-0.910
H	2.838	-6.943	-1.801
C	3.806	-6.872	0.112
N	4.921	-6.063	0.260
H	5.612	-5.840	-0.450
C	5.027	-5.663	1.535
H	5.805	-5.044	1.975
N	4.002	-6.159	2.213
C	3.238	-6.916	1.349
H	2.347	-7.435	1.654
C	7.134	-8.713	3.912
H	6.742	-7.696	3.850
C	6.281	-9.502	4.928
H	6.446	-9.117	5.942
H	6.563	-10.559	4.954
C	4.801	-9.391	4.692
N	3.962	-9.241	5.778
H	4.285	-9.217	6.750
C	2.692	-9.165	5.363
H	1.843	-9.050	6.019
N	2.649	-9.238	4.032
C	3.963	-9.388	3.601
H	4.200	-9.434	2.550
C	2.301	-12.905	-1.731
H	2.296	-13.910	-2.159
C	1.095	-12.737	-0.779
H	1.230	-13.459	0.021
H	0.160	-13.023	-1.270
C	0.924	-11.394	-0.137
N	-0.150	-10.562	-0.379
H	-0.933	-10.725	-1.026
C	-0.063	-9.487	0.418
H	-0.830	-8.734	0.484
N	1.047	-9.556	1.158
C	1.665	-10.747	0.823
H	2.565	-11.064	1.328
C	0.623	-11.386	-4.792
H	1.371	-10.731	-5.241
C	-0.199	-12.109	-5.836
H	-1.008	-12.705	-5.395
H	0.442	-12.784	-6.410
S	-0.970	-10.960	-7.050
C	-1.231	-8.208	-3.522
H	-1.597	-8.660	-2.596
C	-0.455	-6.915	-3.181
H	-0.561	-6.135	-3.944
H	0.624	-7.124	-3.137
C	-0.801	-6.400	-1.825
N	-2.061	-6.025	-1.392
H	-2.905	-5.997	-1.964
C	-1.985	-5.666	-0.090
H	-2.821	-5.323	0.499
N	-0.734	-5.787	0.339
C	0.003	-6.262	-0.724
H	1.047	-6.499	-0.614
C	-5.447	-13.898	-6.167
H	-5.880	-14.789	-6.621
C	-4.261	-13.386	-7.000
H	-3.477	-14.156	-7.059
H	-3.778	-12.524	-6.516
C	-4.604	-13.010	-8.405
N	-3.635	-12.457	-9.228
C	-4.200	-12.233	-10.412
H	-3.716	-11.761	-11.253
N	-5.483	-12.629	-10.386
H	-6.100	-12.635	-11.200
C	-5.760	-13.125	-9.129
H	-6.724	-13.531	-8.869
C	-1.379	-17.021	-4.088
H	-0.858	-17.930	-4.406
C	-1.506	-16.042	-5.268
H	-1.903	-15.073	-4.943
H	-2.246	-16.452	-5.950
C	-0.194	-15.829	-6.035
H	0.271	-16.786	-6.297
H	0.524	-15.274	-5.425
S	-0.351	-14.884	-7.628
C	-0.380	-16.254	-8.827
H	-0.512	-15.824	-9.825
H	-1.190	-16.956	-8.630
H	0.557	-16.808	-8.779
CU	-1.757	-12.166	-8.676
CU	0.923	-8.897	3.096
CU	-0.011	-5.468	2.231
CU	3.810	-6.001	4.092
H	-1.085	-7.756	2.457
O	-0.212	-7.380	2.731
H	5.712	-6.318	5.773
O	5.498	-6.264	4.810
O	-2.636	-8.759	2.297
H	-3.531	-8.480	2.548
H	-2.797	-9.677	1.909
O	6.524	-6.483	7.285
H	5.908	-6.869	7.979
H	6.827	-5.630	7.669
O	6.393	-4.036	3.607
H	6.405	-4.869	4.131
H	7.300	-3.685	3.617
H	-1.688	-6.064	8.974
H	-0.509	-4.855	8.522
H	1.970	-2.641	6.176
H	3.257	-1.931	5.224
H	-4.129	-2.727	6.732
H	-5.072	-4.180	6.638
H	-5.286	-8.946	6.584
H	-5.128	-10.693	6.499
H	-2.894	-12.578	-16.290
H	-2.043	-11.535	-15.215
H	0.523	-9.604	-11.102
H	1.363	-9.128	-9.647
H	4.468	-8.384	-2.769
H	5.379	-8.694	-1.333
H	7.169	-9.124	3.036
H	8.240	-8.607	4.332
H	3.148	-12.706	-1.308
H	2.144	-12.163	-2.625
H	1.029	-12.004	-4.174
H	-0.104	-10.692	-4.170
H	-0.692	-8.844	-4.010
H	-2.219	-7.944	-4.123
H	-6.128	-13.232	-6.004
H	-5.009	-14.252	-5.133
H	-2.240	-17.260	-3.714
H	-0.688	-16.524	-3.266

212
WT FRW

C	-0.812	-6.005	8.610
H	-0.074	-6.447	9.280
C	-0.721	-6.673	7.236
H	-1.483	-6.301	6.543
H	-0.907	-7.747	7.377
C	0.622	-6.452	6.627
N	1.765	-6.420	7.408
H	1.839	-6.850	8.326
C	2.819	-6.122	6.640
H	3.841	-6.090	6.978
N	2.404	-5.900	5.405
C	1.039	-6.098	5.376
H	0.469	-6.003	4.469
C	2.437	-2.772	5.344
H	2.977	-3.716	5.358
C	1.491	-2.745	4.137
H	0.950	-1.794	4.081
H	0.729	-3.518	4.295
C	2.143	-3.043	2.819
N	1.583	-3.950	1.927
C	2.385	-3.990	0.870
H	2.246	-4.617	0.006
N	3.428	-3.148	1.029
H	4.229	-3.004	0.407
C	3.291	-2.537	2.257
H	4.027	-1.829	2.602
C	-4.915	-3.081	6.292
H	-5.825	-2.533	6.545
C	-4.743	-3.100	4.754
H	-4.558	-2.072	4.420
H	-5.684	-3.408	4.280
C	-3.625	-3.959	4.252
N	-3.668	-5.343	4.267
H	-4.438	-5.942	4.580
C	-2.486	-5.807	3.817
H	-2.254	-6.853	3.746
N	-1.678	-4.804	3.494
C	-2.377	-3.648	3.778
H	-1.944	-2.675	3.611
C	-4.741	-9.611	6.186
H	-4.840	-9.589	5.099
C	-3.269	-9.435	6.622
H	-3.085	-9.891	7.601
H	-3.049	-8.365	6.745
C	-2.300	-9.935	5.604
N	-2.406	-11.165	4.994
H	-3.159	-11.836	5.170
C	-1.444	-11.270	4.054
H	-1.350	-12.110	3.393
N	-0.702	-10.172	4.032
C	-1.236	-9.325	4.989
H	-0.816	-8.346	5.145
C	-2.967	-12.246	-15.392
H	-3.857	-11.621	-15.271
C	-2.981	-13.380	-14.335
H	-3.917	-13.921	-14.512
H	-3.087	-12.958	-13.331
C	-1.835	-14.359	-14.345
C	-0.758	-14.381	-13.486
N	0.091	-15.428	-13.772
H	0.964	-15.620	-13.288
C	-0.409	-16.111	-14.848
C	0.079	-17.249	-15.492
H	1.031	-17.687	-15.207
C	-0.715	-17.810	-16.488
H	-0.358	-18.682	-17.024
C	-1.969	-17.261	-16.819
H	-2.593	-17.776	-17.542
C	-2.435	-16.102	-16.204
H	-3.422	-15.711	-16.441
C	-1.646	-15.492	-15.213
C	0.650	-9.900	-10.196
H	-0.284	-9.905	-9.634
C	1.281	-11.313	-10.099
H	1.308	-11.599	-9.041
H	2.300	-11.321	-10.503
C	0.459	-12.327	-10.831
N	-0.747	-12.784	-10.326
C	-1.303	-13.534	-11.246
H	-2.247	-14.050	-11.153
N	-0.508	-13.590	-12.365
C	0.633	-12.839	-12.090
H	1.403	-12.714	-12.833
C	4.711	-7.944	-1.956
H	5.331	-7.070	-2.148
C	3.464	-7.575	-1.150
H	2.847	-8.451	-0.918
H	2.838	-6.923	-1.781
C	3.823	-6.910	0.130
N	4.890	-6.035	0.248
H	5.533	-5.760	-0.488
C	5.075	-5.750	1.552
H	5.861	-5.127	1.950
N	4.159	-6.372	2.266
C	3.375	-7.105	1.403
H	2.582	-7.745	1.750
C	7.136	-8.725	3.921
H	6.727	-7.716	3.870
C	6.314	-9.537	4.942
H	6.486	-9.164	5.959
H	6.618	-10.587	4.947
C	4.838	-9.430	4.708
N	3.988	-9.162	5.759
H	4.285	-9.103	6.736
C	2.724	-9.110	5.298
H	1.856	-8.929	5.915
N	2.708	-9.313	3.986
C	4.022	-9.516	3.607
H	4.282	-9.673	2.571
C	2.314	-12.912	-1.738
H	2.310	-13.916	-2.173
C	1.116	-12.740	-0.782
H	1.262	-13.433	0.043
H	0.183	-13.050	-1.264
C	0.942	-11.367	-0.211
N	-0.181	-10.608	-0.434
H	-0.968	-10.818	-1.053
C	-0.079	-9.467	0.268
H	-0.872	-8.740	0.302
N	1.083	-9.414	0.922
C	1.731	-10.604	0.616
H	2.706	-10.824	1.025
C	0.638	-11.383	-4.792
H	1.390	-10.719	-5.228
C	-0.173	-12.064	-5.878
H	-0.977	-12.665	-5.439
H	0.500	-12.735	-6.421
S	-0.871	-10.810	-7.048
C	-1.223	-8.205	-3.510
H	-1.593	-8.664	-2.591
C	-0.447	-6.916	-3.154
H	-0.529	-6.142	-3.928
H	0.628	-7.135	-3.081
C	-0.829	-6.395	-1.810
N	-2.119	-6.086	-1.425
H	-2.941	-6.165	-2.015
C	-2.094	-5.701	-0.123
H	-2.973	-5.431	0.442
N	-0.856	-5.725	0.344
C	-0.065	-6.181	-0.692
H	0.991	-6.363	-0.559
C	-5.443	-13.892	-6.170
H	-5.875	-14.785	-6.623
C	-4.265	-13.373	-7.010
H	-3.476	-14.137	-7.070
H	-3.774	-12.507	-6.543
C	-4.622	-13.012	-8.417
N	-3.658	-12.468	-9.246
C	-4.231	-12.241	-10.419
H	-3.743	-11.770	-11.259
N	-5.525	-12.621	-10.387
H	-6.142	-12.640	-11.197
C	-5.789	-13.123	-9.127
H	-6.751	-13.526	-8.858
C	-1.379	-17.021	-4.088
H	-0.856	-17.930	-4.407
C	-1.502	-16.043	-5.270
H	-1.894	-15.072	-4.950
H	-2.244	-16.453	-5.951
C	-0.189	-15.833	-6.039
H	0.280	-16.792	-6.288
H	0.520	-15.270	-5.426
S	-0.349	-14.889	-7.622
C	-0.382	-16.261	-8.820
H	-0.516	-15.822	-9.814
H	-1.193	-16.965	-8.630
H	0.555	-16.819	-8.781
CU	-1.769	-12.079	-8.633
CU	0.936	-9.573	3.108
CU	-0.144	-4.999	2.175
CU	3.638	-6.067	4.026
O	-0.118	-7.398	2.650
H	6.121	-5.491	4.550
H	-0.985	-7.824	2.401
O	5.589	-6.175	5.006
H	5.975	-6.297	5.904
H	0.429	-7.517	1.856
O	-2.598	-8.571	2.104
H	-2.947	-8.782	2.987
H	-2.747	-9.427	1.619
O	6.729	-6.577	7.438
H	5.996	-6.935	8.026
H	6.912	-5.688	7.819
O	6.837	-3.989	4.105
H	7.664	-4.084	4.642
H	7.114	-3.433	3.357
H	-1.699	-6.062	8.970
H	-0.514	-4.866	8.504
H	1.969	-2.638	6.174
H	3.257	-1.926	5.228
H	-4.147	-2.722	6.756
H	-5.089	-4.178	6.662
H	-5.298	-8.941	6.585
H	-5.126	-10.684	6.501
H	-2.895	-12.576	-16.292
H	-2.042	-11.537	-15.214
H	0.525	-9.608	-11.107
H	1.363	-9.137	-9.645
H	4.469	-8.384	-2.767
H	5.377	-8.691	-1.330
H	7.173	-9.128	3.040
H	8.243	-8.609	4.332
H	3.161	-12.710	-1.318
H	2.151	-12.168	-2.629
H	1.033	-12.008	-4.177
H	-0.095	-10.693	-4.173
H	-0.675	-8.837	-3.992
H	-2.212	-7.943	-4.115
H	-6.126	-13.229	-6.003
H	-5.006	-14.248	-5.134
H	-2.240	-17.261	-3.714
H	-0.690	-16.520	-3.266

209
WT FR

C	-0.812	-6.003	8.611
H	-0.073	-6.445	9.281
C	-0.726	-6.669	7.238
H	-1.491	-6.299	6.547
H	-0.909	-7.743	7.379
C	0.614	-6.446	6.627
N	1.759	-6.433	7.401
H	1.831	-6.863	8.318
C	2.812	-6.127	6.636
H	3.835	-6.104	6.972
N	2.394	-5.883	5.408
C	1.028	-6.074	5.380
H	0.446	-5.923	4.488
C	2.440	-2.770	5.341
H	2.979	-3.715	5.353
C	1.500	-2.736	4.129
H	0.961	-1.784	4.080
H	0.737	-3.508	4.282
C	2.155	-3.026	2.807
N	1.583	-3.901	1.893
C	2.387	-3.942	0.837
H	2.236	-4.556	-0.034
N	3.446	-3.129	1.019
H	4.254	-2.997	0.404
C	3.317	-2.537	2.257
H	4.068	-1.852	2.613
C	-4.867	-3.107	6.225
H	-5.788	-2.547	6.398
C	-4.603	-3.192	4.705
H	-4.377	-2.181	4.349
H	-5.525	-3.495	4.190
C	-3.490	-4.090	4.254
N	-3.594	-5.470	4.151
H	-4.386	-6.055	4.439
C	-2.437	-5.955	3.646
H	-2.269	-6.997	3.400
N	-1.583	-4.957	3.422
C	-2.229	-3.796	3.806
H	-1.756	-2.830	3.711
C	-4.740	-9.615	6.184
H	-4.839	-9.594	5.097
C	-3.267	-9.445	6.620
H	-3.080	-9.933	7.584
H	-3.051	-8.379	6.780
C	-2.292	-9.896	5.586
N	-2.384	-11.085	4.894
H	-3.125	-11.780	5.014
C	-1.407	-11.122	3.961
H	-1.297	-11.917	3.244
N	-0.668	-10.021	4.029
C	-1.217	-9.251	5.034
H	-0.813	-8.278	5.252
C	-2.966	-12.247	-15.388
H	-3.856	-11.621	-15.265
C	-2.979	-13.380	-14.331
H	-3.916	-13.921	-14.508
H	-3.084	-12.957	-13.328
C	-1.832	-14.359	-14.339
C	-0.753	-14.373	-13.481
N	0.105	-15.411	-13.782
H	0.974	-15.609	-13.291
C	-0.400	-16.105	-14.847
C	0.097	-17.237	-15.495
H	1.047	-17.678	-15.205
C	-0.691	-17.801	-16.495
H	-0.329	-18.672	-17.029
C	-1.949	-17.258	-16.823
H	-2.571	-17.774	-17.548
C	-2.425	-16.106	-16.203
H	-3.413	-15.723	-16.445
C	-1.641	-15.491	-15.209
C	0.649	-9.897	-10.190
H	-0.285	-9.897	-9.631
C	1.279	-11.311	-10.084
H	1.303	-11.600	-9.027
H	2.304	-11.331	-10.476
C	0.451	-12.316	-10.822
N	-0.754	-12.772	-10.314
C	-1.301	-13.530	-11.230
H	-2.241	-14.059	-11.137
N	-0.508	-13.590	-12.354
C	0.626	-12.824	-12.084
H	1.394	-12.687	-12.825
C	4.708	-7.947	-1.960
H	5.326	-7.071	-2.153
C	3.454	-7.583	-1.167
H	2.845	-8.462	-0.931
H	2.827	-6.947	-1.813
C	3.792	-6.892	0.105
N	4.910	-6.086	0.240
H	5.584	-5.855	-0.482
C	5.049	-5.743	1.534
H	5.863	-5.165	1.940
N	4.053	-6.258	2.223
C	3.260	-6.980	1.358
H	2.394	-7.533	1.685
C	7.133	-8.715	3.916
H	6.737	-7.702	3.856
C	6.284	-9.505	4.935
H	6.459	-9.128	5.951
H	6.566	-10.562	4.952
C	4.809	-9.369	4.696
N	3.954	-9.179	5.762
H	4.251	-9.160	6.740
C	2.692	-9.085	5.300
H	1.823	-8.943	5.925
N	2.680	-9.185	3.978
C	3.995	-9.368	3.588
H	4.258	-9.440	2.544
C	2.278	-12.884	-1.704
H	2.244	-13.898	-2.109
C	1.075	-12.646	-0.765
H	1.180	-13.327	0.080
H	0.143	-12.937	-1.259
C	0.919	-11.257	-0.214
N	-0.217	-10.494	-0.387
H	-1.035	-10.693	-0.974
C	-0.113	-9.377	0.356
H	-0.920	-8.671	0.477
N	1.066	-9.336	0.979
C	1.716	-10.512	0.619
H	2.692	-10.744	1.021
C	0.653	-11.366	-4.786
H	1.407	-10.691	-5.192
C	-0.120	-12.035	-5.905
H	-0.922	-12.673	-5.522
H	0.577	-12.664	-6.472
S	-0.835	-10.773	-7.058
C	-1.229	-8.197	-3.514
H	-1.593	-8.655	-2.590
C	-0.458	-6.901	-3.167
H	-0.617	-6.103	-3.904
H	0.624	-7.089	-3.175
C	-0.782	-6.441	-1.784
N	-2.068	-6.201	-1.331
H	-2.908	-6.275	-1.899
C	-2.014	-5.918	-0.008
H	-2.876	-5.713	0.607
N	-0.757	-5.943	0.416
C	0.012	-6.282	-0.679
H	1.072	-6.444	-0.577
C	-5.443	-13.895	-6.171
H	-5.877	-14.787	-6.624
C	-4.265	-13.374	-7.012
H	-3.479	-14.140	-7.086
H	-3.772	-12.518	-6.531
C	-4.629	-12.998	-8.413
N	-3.680	-12.439	-9.252
C	-4.268	-12.233	-10.419
H	-3.800	-11.770	-11.276
N	-5.557	-12.631	-10.374
H	-6.177	-12.660	-11.179
C	-5.802	-13.125	-9.108
H	-6.756	-13.536	-8.823
C	-1.376	-17.023	-4.089
H	-0.852	-17.934	-4.401
C	-1.492	-16.050	-5.274
H	-1.887	-15.079	-4.957
H	-2.229	-16.461	-5.960
C	-0.173	-15.840	-6.035
H	0.299	-16.800	-6.278
H	0.531	-15.274	-5.418
S	-0.333	-14.893	-7.618
C	-0.380	-16.267	-8.812
H	-0.523	-15.830	-9.807
H	-1.190	-16.972	-8.618
H	0.555	-16.829	-8.786
CU	-1.850	-11.970	-8.599
CU	0.897	-9.397	3.015
CU	-0.079	-4.999	2.086
CU	3.602	-6.006	4.005
O	5.560	-6.192	4.973
H	6.104	-5.500	4.545
H	5.930	-6.326	5.878
O	-2.934	-8.623	2.221
H	-3.535	-8.784	2.966
H	-2.940	-9.515	1.777
O	6.707	-6.576	7.414
H	5.984	-6.939	8.011
H	6.900	-5.691	7.799
O	6.827	-3.998	4.144
H	7.659	-4.090	4.678
H	7.106	-3.467	3.378
H	-1.698	-6.059	8.973
H	-0.513	-4.866	8.505
H	1.971	-2.639	6.171
H	3.258	-1.924	5.228
H	-4.127	-2.731	6.719
H	-5.067	-4.189	6.628
H	-5.294	-8.942	6.582
H	-5.126	-10.686	6.498
H	-2.896	-12.578	-16.288
H	-2.041	-11.537	-15.213
H	0.522	-9.608	-11.102
H	1.363	-9.133	-9.644
H	4.470	-8.387	-2.771
H	5.374	-8.693	-1.333
H	7.171	-9.123	3.039
H	8.240	-8.608	4.333
H	3.130	-12.695	-1.290
H	2.137	-12.157	-2.614
H	1.046	-12.001	-4.178
H	-0.093	-10.683	-4.170
H	-0.687	-8.831	-3.998
H	-2.219	-7.942	-4.119
H	-6.126	-13.231	-6.004
H	-5.001	-14.248	-5.138
H	-2.238	-17.262	-3.715
H	-0.688	-16.522	-3.266

213

M467Q NI

C	-1.799	-2.196	-7.674
H	-2.417	-2.886	-8.251
C	-0.320	-2.549	-7.820
H	0.317	-1.862	-7.251
H	-0.040	-2.428	-8.871
C	-0.051	-3.930	-7.339
N	-0.926	-4.986	-7.517
H	-1.670	-5.025	-8.206
C	-0.473	-6.058	-6.846
H	-0.952	-7.021	-6.823
N	0.646	-5.733	-6.226
C	0.917	-4.412	-6.513
H	1.742	-3.873	-6.091
C	-0.257	-5.408	-3.284
H	-0.101	-6.105	-4.104
C	1.054	-4.657	-2.975
H	0.938	-4.010	-2.100
H	1.258	-3.984	-3.816
C	2.275	-5.516	-2.804
N	3.504	-5.227	-3.400
C	4.345	-6.185	-3.019
H	5.370	-6.267	-3.332
N	3.724	-7.073	-2.222
H	4.093	-7.941	-1.823
C	2.412	-6.687	-2.097
H	1.729	-7.247	-1.485
C	0.401	1.766	-4.612
H	0.206	2.787	-4.284
C	1.778	1.303	-4.065
H	1.702	1.247	-2.973
H	2.534	2.072	-4.265
C	2.293	-0.011	-4.573
N	2.828	-0.112	-5.841
H	2.884	0.612	-6.559
C	3.197	-1.388	-6.061
H	3.648	-1.733	-6.969
N	2.984	-2.131	-4.976
C	2.405	-1.278	-4.046
H	2.106	-1.629	-3.071
C	2.687	0.574	-10.658
H	3.714	0.547	-10.290
C	1.992	-0.797	-10.477
H	1.258	-0.963	-11.273
H	1.416	-0.797	-9.542
C	2.902	-1.977	-10.342
N	3.973	-2.314	-11.150
H	4.369	-1.741	-11.898
C	4.519	-3.465	-10.686
H	5.411	-3.909	-11.091
N	3.848	-3.892	-9.622
C	2.857	-2.961	-9.393
H	2.175	-3.081	-8.572
C	23.372	-4.887	-5.135
H	23.199	-3.956	-4.592
C	22.892	-4.760	-6.598
H	23.389	-3.872	-7.008
H	21.821	-4.528	-6.638
C	23.203	-5.955	-7.468
C	22.600	-7.180	-7.379
H	21.820	-7.517	-6.713
N	23.106	-8.040	-8.340
H	22.892	-9.028	-8.329
C	24.096	-7.389	-9.053
C	24.883	-7.811	-10.132
H	24.777	-8.812	-10.548
C	25.769	-6.889	-10.691
H	26.328	-7.151	-11.588
C	25.886	-5.582	-10.168
H	26.583	-4.890	-10.631
C	25.115	-5.172	-9.085
H	25.197	-4.157	-8.703
C	24.190	-6.066	-8.517
C	16.840	-7.322	-4.742
H	16.526	-6.321	-5.028
C	16.965	-8.188	-6.015
H	16.033	-8.066	-6.582
H	17.079	-9.253	-5.770
C	18.079	-7.790	-6.934
N	18.139	-6.531	-7.491
C	19.150	-6.521	-8.330
H	19.457	-5.672	-8.923
N	19.760	-7.741	-8.355
H	20.552	-8.017	-8.918
C	19.088	-8.563	-7.462
H	19.385	-9.588	-7.305
C	7.821	-9.784	-5.382
H	7.594	-10.327	-4.464
C	7.198	-8.383	-5.361
H	7.405	-7.844	-6.294
H	7.696	-7.805	-4.570
C	5.713	-8.415	-5.190
N	5.028	-9.230	-4.304
H	5.448	-9.892	-3.661
C	3.703	-9.162	-4.552
H	2.935	-9.757	-4.069
N	3.496	-8.305	-5.537
C	4.737	-7.846	-5.950
H	4.870	-7.202	-6.796
C	2.004	-11.060	-7.905
H	1.806	-10.548	-6.958
C	1.507	-10.218	-9.103
H	0.415	-10.126	-9.076
H	1.752	-10.698	-10.056
C	2.034	-8.814	-9.068
N	1.155	-7.754	-9.183
H	0.168	-7.847	-9.445
C	1.845	-6.609	-9.037
H	1.422	-5.628	-8.999
N	3.126	-6.861	-8.817
C	3.263	-8.239	-8.845
H	4.213	-8.710	-8.653
C	9.694	-8.151	-10.307
H	10.413	-8.376	-11.099
C	9.024	-6.787	-10.588
H	8.527	-6.880	-11.550
H	9.777	-6.004	-10.727
C	8.006	-6.317	-9.594
N	8.269	-5.308	-8.692
H	9.137	-4.769	-8.633
C	7.157	-5.073	-7.972
H	7.071	-4.285	-7.247
N	6.180	-5.895	-8.336
C	6.701	-6.676	-9.352
H	6.088	-7.410	-9.854
C	12.333	-6.797	-7.946
H	12.221	-7.438	-7.070
C	13.816	-6.524	-8.144
H	14.060	-6.002	-9.066
H	14.303	-7.510	-8.220
S	14.554	-5.619	-6.724
C	10.472	-4.149	-5.756
H	9.858	-3.709	-6.547
C	9.560	-4.690	-4.629
H	10.043	-4.638	-3.645
H	9.363	-5.760	-4.790
C	8.211	-4.055	-4.593
N	7.950	-2.700	-4.515
H	8.653	-1.969	-4.440
C	6.607	-2.514	-4.528
H	6.125	-1.550	-4.518
N	5.990	-3.683	-4.601
C	6.977	-4.645	-4.662
H	6.738	-5.684	-4.816
C	15.615	-1.348	-10.424
H	16.390	-1.154	-11.161
C	15.978	-2.543	-9.525
H	16.049	-3.468	-10.111
H	15.194	-2.750	-8.790
C	17.251	-2.412	-8.757
N	17.639	-3.438	-7.909
C	18.774	-3.068	-7.340
H	19.302	-3.621	-6.578
N	19.137	-1.842	-7.778
H	20.021	-1.390	-7.561
C	18.184	-1.416	-8.686
H	18.260	-0.491	-9.230
C	13.800	-5.442	-13.727
H	14.257	-6.120	-14.457
C	14.663	-5.386	-12.463
H	14.205	-4.797	-11.667
H	15.567	-4.854	-12.750
C	15.081	-6.761	-11.880
H	15.237	-7.486	-12.690
H	14.281	-7.148	-11.241
C	16.338	-6.641	-11.041
O	16.450	-5.866	-10.099
N	17.402	-7.434	-11.427
H	18.301	-7.141	-11.070
H	17.407	-7.826	-12.366
CU	16.532	-5.044	-7.526
CU	4.108	-5.222	-8.040
CU	4.047	-4.186	-5.147
CU	1.923	-7.111	-5.699
H	-0.044	-8.524	-5.728
O	3.280	-5.727	-6.197
H	5.237	-3.083	-7.167
O	0.504	-8.174	-4.990
O	4.466	-3.692	-6.954
O	-1.467	-9.362	-6.973
H	-2.164	-9.494	-6.302
H	-1.902	-8.775	-7.650
O	1.238	-10.613	-4.386
H	0.908	-9.695	-4.554
H	0.435	-11.138	-4.199
O	6.213	-1.843	-7.587
H	5.918	-1.648	-8.506
H	7.176	-2.061	-7.838
H	-1.950	-1.284	-7.925
H	-2.097	-2.344	-6.539
H	-0.969	-4.790	-3.498
H	-0.581	-6.092	-2.366
H	-0.312	1.175	-4.340
H	0.439	1.826	-5.790
H	2.197	1.270	-10.208
H	2.781	0.816	-11.815
H	24.292	-5.156	-5.087
H	22.732	-5.713	-4.575
H	17.653	-7.309	-4.227
H	15.962	-7.778	-4.085
H	8.766	-9.743	-5.536
H	7.318	-10.389	-6.262
H	2.942	-11.294	-7.974
H	1.360	-12.058	-7.862
H	9.049	-8.861	-10.206
H	10.322	-8.038	-9.314
H	11.929	-7.203	-8.718
H	11.720	-5.807	-7.684
H	11.031	-4.845	-6.123
H	11.125	-3.244	-5.345
H	15.412	-0.544	-9.928
H	14.664	-1.657	-11.061
H	13.680	-4.570	-14.136
H	12.758	-5.933	-13.457

214
M467Q NIH+

C	-1.799	-2.194	-7.675
H	-2.414	-2.884	-8.252
C	-0.317	-2.535	-7.819
H	0.314	-1.838	-7.259
H	-0.046	-2.420	-8.872
C	-0.037	-3.911	-7.326
N	-0.902	-4.974	-7.523
H	-1.620	-5.015	-8.240
C	-0.437	-6.048	-6.870
H	-0.918	-7.008	-6.872
N	0.676	-5.723	-6.232
C	0.931	-4.389	-6.500
H	1.774	-3.861	-6.098
C	-0.253	-5.400	-3.275
H	-0.070	-6.080	-4.104
C	1.044	-4.633	-2.947
H	0.899	-3.989	-2.075
H	1.258	-3.959	-3.784
C	2.265	-5.478	-2.750
N	3.486	-5.192	-3.353
C	4.334	-6.139	-2.967
H	5.358	-6.227	-3.286
N	3.725	-7.014	-2.137
H	4.102	-7.872	-1.728
C	2.416	-6.616	-1.993
H	1.732	-7.169	-1.371
C	0.408	1.760	-4.609
H	0.216	2.782	-4.278
C	1.789	1.305	-4.048
H	1.702	1.240	-2.957
H	2.535	2.088	-4.233
C	2.334	0.003	-4.556
N	2.904	-0.071	-5.809
H	2.968	0.667	-6.515
C	3.315	-1.326	-6.031
H	3.773	-1.642	-6.949
N	3.066	-2.101	-4.977
C	2.452	-1.273	-4.049
H	2.161	-1.643	-3.079
C	2.687	0.574	-10.662
H	3.712	0.541	-10.296
C	1.985	-0.793	-10.491
H	1.252	-0.961	-11.288
H	1.401	-0.801	-9.560
C	2.894	-1.965	-10.367
N	3.969	-2.296	-11.154
H	4.388	-1.716	-11.887
C	4.510	-3.450	-10.681
H	5.418	-3.879	-11.064
N	3.821	-3.892	-9.633
C	2.846	-2.963	-9.411
H	2.153	-3.066	-8.598
C	23.371	-4.886	-5.128
H	23.199	-3.952	-4.588
C	22.894	-4.754	-6.594
H	23.389	-3.864	-7.001
H	21.822	-4.521	-6.636
C	23.208	-5.945	-7.467
C	22.632	-7.182	-7.370
H	21.879	-7.536	-6.681
N	23.119	-8.027	-8.357
H	22.951	-9.024	-8.324
C	24.101	-7.358	-9.071
C	24.861	-7.758	-10.178
H	24.758	-8.754	-10.603
C	25.743	-6.827	-10.727
H	26.299	-7.076	-11.628
C	25.865	-5.529	-10.184
H	26.553	-4.825	-10.643
C	25.086	-5.127	-9.104
H	25.184	-4.123	-8.699
C	24.176	-6.036	-8.536
C	16.839	-7.321	-4.747
H	16.519	-6.316	-5.022
C	16.970	-8.193	-6.020
H	16.042	-8.094	-6.595
H	17.091	-9.254	-5.769
C	18.086	-7.797	-6.940
N	18.139	-6.546	-7.524
C	19.173	-6.533	-8.343
H	19.480	-5.683	-8.935
N	19.797	-7.738	-8.328
H	20.636	-7.990	-8.833
C	19.135	-8.546	-7.421
H	19.446	-9.564	-7.243
C	7.836	-9.797	-5.384
H	7.605	-10.349	-4.472
C	7.257	-8.384	-5.344
H	7.463	-7.845	-6.277
H	7.785	-7.824	-4.558
C	5.785	-8.386	-5.150
N	5.111	-9.112	-4.179
H	5.526	-9.778	-3.533
C	3.788	-9.031	-4.425
H	3.024	-9.509	-3.834
N	3.575	-8.260	-5.479
C	4.817	-7.866	-5.948
H	4.956	-7.217	-6.790
C	1.999	-11.065	-7.941
H	1.785	-10.512	-7.024
C	1.521	-10.240	-9.160
H	0.427	-10.165	-9.170
H	1.799	-10.728	-10.098
C	2.033	-8.832	-9.105
N	1.150	-7.776	-9.229
H	0.169	-7.865	-9.514
C	1.832	-6.630	-9.050
H	1.405	-5.652	-9.014
N	3.108	-6.878	-8.812
C	3.254	-8.253	-8.843
H	4.204	-8.721	-8.638
C	9.677	-8.134	-10.306
H	10.395	-8.345	-11.104
C	8.989	-6.772	-10.576
H	8.485	-6.873	-11.534
H	9.734	-5.984	-10.729
C	7.974	-6.301	-9.580
N	8.218	-5.254	-8.717
H	9.070	-4.690	-8.676
C	7.098	-5.010	-8.008
H	7.004	-4.190	-7.320
N	6.136	-5.860	-8.344
C	6.672	-6.667	-9.328
H	6.070	-7.426	-9.807
C	12.335	-6.792	-7.947
H	12.232	-7.432	-7.069
C	13.811	-6.510	-8.172
H	14.056	-6.011	-9.107
H	14.322	-7.484	-8.215
S	14.597	-5.555	-6.817
C	10.472	-4.144	-5.763
H	9.865	-3.702	-6.558
C	9.552	-4.679	-4.641
H	10.025	-4.613	-3.653
H	9.362	-5.751	-4.795
C	8.202	-4.047	-4.628
N	7.939	-2.694	-4.527
H	8.637	-1.959	-4.433
C	6.595	-2.512	-4.571
H	6.106	-1.553	-4.564
N	5.981	-3.679	-4.685
C	6.970	-4.637	-4.743
H	6.736	-5.672	-4.955
C	15.609	-1.349	-10.430
H	16.393	-1.159	-11.163
C	15.982	-2.545	-9.536
H	16.044	-3.468	-10.126
H	15.197	-2.749	-8.798
C	17.257	-2.432	-8.769
N	17.654	-3.484	-7.958
C	18.789	-3.126	-7.372
H	19.322	-3.702	-6.629
N	19.139	-1.886	-7.768
H	20.013	-1.430	-7.514
C	18.181	-1.431	-8.656
H	18.251	-0.491	-9.176
C	13.798	-5.443	-13.725
H	14.257	-6.125	-14.452
C	14.663	-5.392	-12.461
H	14.204	-4.803	-11.664
H	15.572	-4.865	-12.744
C	15.073	-6.773	-11.882
H	15.244	-7.488	-12.696
H	14.260	-7.170	-11.264
C	16.318	-6.652	-11.027
O	16.384	-5.937	-10.024
N	17.403	-7.388	-11.439
H	18.301	-7.088	-11.069
H	17.424	-7.760	-12.387
CU	16.596	-5.154	-7.651
CU	4.018	-5.232	-7.984
CU	3.989	-4.147	-5.174
CU	2.045	-7.025	-5.769
H	-0.128	-8.627	-5.892
O	3.393	-5.728	-6.154
O	0.613	-8.448	-5.257
H	0.982	-9.352	-5.061
H	5.197	-3.150	-7.184
O	4.363	-3.639	-6.997
O	-1.508	-9.235	-6.869
H	-2.254	-9.362	-6.240
H	-1.916	-8.683	-7.600
O	1.360	-10.900	-4.531
H	1.993	-11.550	-4.898
H	0.535	-11.368	-4.287
O	6.190	-1.860	-7.658
H	5.921	-1.781	-8.603
H	7.174	-2.004	-7.860
H	-1.951	-1.284	-7.925
H	-2.098	-2.344	-6.541
H	-0.968	-4.790	-3.494
H	-0.581	-6.090	-2.360
H	-0.307	1.171	-4.340
H	0.447	1.822	-5.787
H	2.199	1.270	-10.210
H	2.781	0.817	-11.818
H	24.292	-5.153	-5.083
H	22.733	-5.713	-4.573
H	17.652	-7.308	-4.231
H	15.964	-7.780	-4.090
H	8.775	-9.764	-5.545
H	7.324	-10.391	-6.269
H	2.939	-11.297	-7.989
H	1.356	-12.061	-7.883
H	9.042	-8.854	-10.207
H	10.312	-8.029	-9.316
H	11.927	-7.198	-8.717
H	11.715	-5.808	-7.685
H	11.035	-4.843	-6.126
H	11.126	-3.240	-5.349
H	15.413	-0.544	-9.931
H	14.661	-1.652	-11.067
H	13.685	-4.570	-14.135
H	12.752	-5.924	-13.456

212
M467Q RO0

C	-1.812	-2.192	-7.675
H	-2.430	-2.880	-8.251
C	-0.335	-2.527	-7.831
H	0.301	-1.827	-7.275
H	-0.064	-2.418	-8.886
C	-0.044	-3.892	-7.337
N	-0.874	-4.983	-7.530
H	-1.610	-5.039	-8.230
C	-0.378	-6.037	-6.870
H	-0.838	-7.009	-6.865
N	0.730	-5.671	-6.236
C	0.930	-4.331	-6.502
H	1.732	-3.760	-6.080
C	-0.264	-5.405	-3.270
H	-0.078	-6.083	-4.100
C	1.029	-4.654	-2.917
H	0.878	-4.011	-2.044
H	1.265	-3.976	-3.747
C	2.234	-5.520	-2.706
N	3.453	-5.235	-3.306
C	4.309	-6.174	-2.915
H	5.334	-6.262	-3.234
N	3.699	-7.044	-2.086
H	4.079	-7.896	-1.665
C	2.387	-6.654	-1.946
H	1.707	-7.213	-1.323
C	0.438	1.726	-4.614
H	0.288	2.744	-4.257
C	1.817	1.220	-4.108
H	1.761	1.142	-3.016
H	2.579	1.985	-4.303
C	2.354	-0.087	-4.629
N	2.959	-0.183	-5.873
H	2.996	0.546	-6.596
C	3.474	-1.410	-6.028
H	4.067	-1.719	-6.877
N	3.228	-2.148	-4.938
C	2.522	-1.329	-4.063
H	2.213	-1.678	-3.091
C	2.678	0.572	-10.661
H	3.704	0.541	-10.293
C	1.975	-0.796	-10.487
H	1.282	-0.988	-11.313
H	1.352	-0.789	-9.582
C	2.892	-1.962	-10.295
N	3.968	-2.310	-11.092
H	4.348	-1.750	-11.864
C	4.555	-3.416	-10.587
H	5.455	-3.854	-10.985
N	3.896	-3.818	-9.499
C	2.869	-2.907	-9.305
H	2.170	-3.023	-8.496
C	23.371	-4.885	-5.127
H	23.203	-3.953	-4.584
C	22.896	-4.755	-6.590
H	23.391	-3.865	-6.995
H	21.825	-4.523	-6.631
C	23.212	-5.943	-7.467
C	22.649	-7.183	-7.364
H	21.922	-7.550	-6.657
N	23.126	-8.021	-8.365
H	22.996	-9.023	-8.316
C	24.102	-7.343	-9.082
C	24.874	-7.739	-10.182
H	24.777	-8.734	-10.613
C	25.737	-6.795	-10.740
H	26.296	-7.041	-11.642
C	25.838	-5.493	-10.205
H	26.522	-4.784	-10.661
C	25.069	-5.104	-9.115
H	25.153	-4.097	-8.714
C	24.170	-6.023	-8.547
C	16.834	-7.320	-4.747
H	16.520	-6.313	-5.010
C	16.968	-8.184	-6.024
H	16.040	-8.107	-6.599
H	17.100	-9.241	-5.769
C	18.083	-7.782	-6.945
N	18.140	-6.534	-7.538
C	19.184	-6.519	-8.345
H	19.496	-5.671	-8.934
N	19.810	-7.718	-8.312
H	20.662	-7.964	-8.795
C	19.136	-8.527	-7.415
H	19.451	-9.539	-7.226
C	7.840	-9.806	-5.381
H	7.615	-10.360	-4.470
C	7.270	-8.393	-5.337
H	7.475	-7.850	-6.268
H	7.795	-7.834	-4.551
C	5.800	-8.373	-5.143
N	5.084	-9.131	-4.230
H	5.465	-9.836	-3.604
C	3.769	-8.934	-4.436
H	2.983	-9.422	-3.882
N	3.612	-8.067	-5.428
C	4.867	-7.731	-5.888
H	5.026	-7.052	-6.700
C	2.000	-11.070	-7.946
H	1.780	-10.515	-7.031
C	1.517	-10.255	-9.168
H	0.422	-10.192	-9.182
H	1.804	-10.738	-10.105
C	2.013	-8.844	-9.105
N	1.125	-7.795	-9.241
H	0.149	-7.893	-9.541
C	1.793	-6.645	-9.041
H	1.358	-5.670	-9.019
N	3.066	-6.883	-8.773
C	3.223	-8.258	-8.811
H	4.174	-8.721	-8.596
C	9.663	-8.121	-10.298
H	10.372	-8.317	-11.106
C	8.959	-6.769	-10.554
H	8.444	-6.873	-11.507
H	9.696	-5.975	-10.718
C	7.942	-6.283	-9.560
N	8.194	-5.207	-8.729
H	9.046	-4.639	-8.715
C	7.077	-4.906	-8.049
H	6.948	-4.039	-7.427
N	6.103	-5.751	-8.365
C	6.629	-6.614	-9.316
H	6.012	-7.370	-9.780
C	12.345	-6.788	-7.954
H	12.248	-7.428	-7.079
C	13.815	-6.498	-8.207
H	14.044	-6.025	-9.159
H	14.335	-7.465	-8.231
S	14.636	-5.514	-6.898
C	10.475	-4.146	-5.769
H	9.877	-3.697	-6.567
C	9.527	-4.676	-4.656
H	9.981	-4.613	-3.659
H	9.338	-5.748	-4.815
C	8.168	-4.050	-4.675
N	7.888	-2.698	-4.556
H	8.566	-1.961	-4.367
C	6.553	-2.519	-4.685
H	6.058	-1.562	-4.646
N	5.951	-3.687	-4.877
C	6.948	-4.643	-4.877
H	6.723	-5.679	-5.070
C	15.617	-1.347	-10.434
H	16.397	-1.152	-11.165
C	15.981	-2.547	-9.541
H	16.032	-3.469	-10.133
H	15.194	-2.734	-8.803
C	17.263	-2.448	-8.783
N	17.653	-3.498	-7.962
C	18.795	-3.148	-7.377
H	19.335	-3.726	-6.643
N	19.158	-1.921	-7.793
H	20.032	-1.462	-7.542
C	18.201	-1.461	-8.679
H	18.280	-0.519	-9.190
C	13.798	-5.442	-13.724
H	14.254	-6.117	-14.455
C	14.661	-5.388	-12.461
H	14.201	-4.798	-11.667
H	15.569	-4.861	-12.747
C	15.072	-6.770	-11.886
H	15.252	-7.476	-12.706
H	14.255	-7.176	-11.280
C	16.307	-6.660	-11.019
O	16.352	-5.980	-9.988
N	17.402	-7.355	-11.453
H	18.297	-7.099	-11.054
H	17.421	-7.756	-12.385
CU	16.640	-5.181	-7.701
CU	4.109	-5.167	-8.080
CU	3.917	-4.043	-5.009
CU	2.150	-6.882	-5.804
H	-0.048	-8.539	-5.920
O	3.509	-5.394	-6.281
H	1.061	-9.276	-5.103
O	0.732	-8.364	-5.330
O	-1.480	-9.141	-6.824
H	-2.222	-9.322	-6.204
H	-1.922	-8.636	-7.573
O	1.340	-10.855	-4.549
H	1.959	-11.534	-4.886
H	0.507	-11.306	-4.291
O	6.067	-2.046	-7.650
H	5.773	-1.964	-8.580
H	7.052	-2.062	-7.854
H	-1.962	-1.281	-7.920
H	-2.105	-2.342	-6.540
H	-0.974	-4.790	-3.493
H	-0.593	-6.093	-2.358
H	-0.289	1.153	-4.337
H	0.456	1.811	-5.791
H	2.192	1.270	-10.207
H	2.775	0.815	-11.818
H	24.293	-5.157	-5.081
H	22.729	-5.711	-4.570
H	17.651	-7.307	-4.231
H	15.962	-7.782	-4.088
H	8.779	-9.772	-5.547
H	7.325	-10.397	-6.264
H	2.941	-11.300	-7.989
H	1.356	-12.063	-7.883
H	9.032	-8.846	-10.204
H	10.306	-8.020	-9.313
H	11.930	-7.198	-8.723
H	11.721	-5.806	-7.690
H	11.043	-4.846	-6.132
H	11.124	-3.241	-5.349
H	15.414	-0.541	-9.932
H	14.667	-1.654	-11.070
H	13.680	-4.566	-14.135
H	12.753	-5.928	-13.457

214

M467Q FRW

C	-1.777	-2.226	-7.678
H	-2.381	-2.928	-8.253
C	-0.281	-2.559	-7.782
H	0.326	-1.852	-7.205
H	0.022	-2.441	-8.828
C	0.016	-3.937	-7.288
N	-0.828	-5.005	-7.538
H	-1.532	-5.033	-8.268
C	-0.368	-6.092	-6.894
H	-0.833	-7.062	-6.937
N	0.721	-5.776	-6.216
C	0.967	-4.433	-6.440
H	1.769	-3.913	-5.941
C	-0.258	-5.420	-3.273
H	-0.077	-6.107	-4.095
C	1.038	-4.675	-2.917
H	0.891	-4.036	-2.041
H	1.281	-3.995	-3.742
C	2.242	-5.547	-2.716
N	3.471	-5.235	-3.285
C	4.321	-6.183	-2.914
H	5.353	-6.248	-3.213
N	3.704	-7.086	-2.127
H	4.081	-7.953	-1.737
C	2.388	-6.706	-1.993
H	1.702	-7.303	-1.415
C	0.362	1.831	-4.619
H	0.112	2.852	-4.325
C	1.756	1.455	-4.057
H	1.682	1.409	-2.964
H	2.474	2.256	-4.271
C	2.333	0.161	-4.542
N	3.034	0.078	-5.729
H	3.109	0.795	-6.457
C	3.452	-1.190	-5.901
H	4.077	-1.496	-6.727
N	3.061	-1.947	-4.880
C	2.356	-1.114	-4.031
H	1.943	-1.482	-3.104
C	2.665	0.563	-10.657
H	3.689	0.491	-10.289
C	1.908	-0.774	-10.483
H	1.123	-0.880	-11.240
H	1.391	-0.781	-9.513
C	2.813	-1.957	-10.452
N	3.849	-2.160	-11.335
H	4.136	-1.510	-12.072
C	4.546	-3.247	-10.939
H	5.461	-3.565	-11.403
N	3.998	-3.783	-9.857
C	2.920	-2.975	-9.541
H	2.250	-3.217	-8.731
C	23.366	-4.882	-5.140
H	23.190	-3.949	-4.601
C	22.884	-4.759	-6.605
H	23.377	-3.870	-7.015
H	21.812	-4.531	-6.643
C	23.194	-5.953	-7.475
C	22.588	-7.177	-7.389
H	21.802	-7.513	-6.730
N	23.094	-8.037	-8.348
H	22.870	-9.024	-8.342
C	24.089	-7.392	-9.057
C	24.872	-7.816	-10.136
H	24.761	-8.815	-10.552
C	25.765	-6.898	-10.692
H	26.323	-7.162	-11.589
C	25.880	-5.590	-10.172
H	26.568	-4.893	-10.641
C	25.102	-5.173	-9.098
H	25.186	-4.158	-8.718
C	24.181	-6.067	-8.525
C	16.834	-7.315	-4.748
H	16.507	-6.317	-5.031
C	16.960	-8.180	-6.021
H	16.031	-8.049	-6.587
H	17.069	-9.245	-5.779
C	18.075	-7.789	-6.939
N	18.149	-6.522	-7.479
C	19.145	-6.523	-8.335
H	19.452	-5.670	-8.922
N	19.743	-7.749	-8.377
H	20.525	-8.030	-8.947
C	19.082	-8.561	-7.469
H	19.363	-9.594	-7.328
C	7.809	-9.750	-5.384
H	7.562	-10.270	-4.459
C	7.239	-8.321	-5.410
H	7.455	-7.821	-6.361
H	7.766	-7.737	-4.639
C	5.760	-8.282	-5.201
N	5.111	-9.149	-4.335
H	5.539	-9.870	-3.761
C	3.781	-8.975	-4.458
H	3.034	-9.540	-3.923
N	3.541	-8.023	-5.343
C	4.761	-7.579	-5.819
H	4.835	-6.804	-6.564
C	1.985	-11.066	-7.918
H	1.760	-10.517	-7.001
C	1.498	-10.257	-9.141
H	0.403	-10.181	-9.143
H	1.773	-10.756	-10.075
C	2.021	-8.860	-9.093
N	1.165	-7.781	-9.198
H	0.180	-7.847	-9.472
C	1.890	-6.655	-9.023
H	1.496	-5.658	-9.026
N	3.160	-6.946	-8.802
C	3.259	-8.319	-8.843
H	4.204	-8.809	-8.662
C	9.779	-8.207	-10.326
H	10.518	-8.478	-11.083
C	9.167	-6.826	-10.644
H	8.701	-6.909	-11.621
H	9.939	-6.060	-10.754
C	8.147	-6.363	-9.659
N	8.366	-5.331	-8.776
H	9.213	-4.759	-8.715
C	7.284	-5.200	-7.988
H	7.183	-4.431	-7.245
N	6.363	-6.107	-8.303
C	6.897	-6.836	-9.347
H	6.327	-7.627	-9.813
C	12.340	-6.799	-7.909
H	12.223	-7.419	-7.019
C	13.820	-6.520	-8.108
H	14.054	-6.008	-9.038
H	14.305	-7.507	-8.176
S	14.542	-5.592	-6.699
C	10.453	-4.123	-5.792
H	9.861	-3.662	-6.587
C	9.518	-4.677	-4.695
H	10.008	-4.714	-3.713
H	9.262	-5.721	-4.931
C	8.212	-3.965	-4.617
N	8.077	-2.600	-4.481
H	8.849	-1.943	-4.413
C	6.754	-2.300	-4.465
H	6.367	-1.297	-4.376
N	6.031	-3.400	-4.581
C	6.928	-4.441	-4.696
H	6.589	-5.453	-4.866
C	15.617	-1.349	-10.433
H	16.399	-1.163	-11.166
C	15.981	-2.540	-9.526
H	16.051	-3.467	-10.108
H	15.195	-2.747	-8.793
C	17.256	-2.406	-8.762
N	17.642	-3.423	-7.904
C	18.776	-3.051	-7.338
H	19.305	-3.605	-6.577
N	19.142	-1.829	-7.787
H	20.021	-1.376	-7.562
C	18.185	-1.409	-8.694
H	18.256	-0.482	-9.236
C	13.805	-5.467	-13.738
H	14.256	-6.154	-14.464
C	14.673	-5.404	-12.477
H	14.221	-4.800	-11.689
H	15.579	-4.881	-12.774
C	15.088	-6.770	-11.875
H	15.229	-7.513	-12.669
H	14.292	-7.137	-11.218
C	16.356	-6.637	-11.051
O	16.481	-5.847	-10.126
N	17.400	-7.478	-11.407
H	18.303	-7.171	-11.063
H	17.407	-7.863	-12.347
CU	16.512	-4.989	-7.490
CU	4.501	-5.417	-8.816
CU	3.969	-3.721	-4.496
CU	1.891	-7.150	-5.617
O	0.237	-8.641	-4.937
H	0.707	-9.496	-4.801
H	-0.389	-8.836	-5.670
O	6.021	-2.281	-7.722
H	5.831	-2.206	-8.685
H	7.019	-2.280	-7.813
O	1.519	-11.032	-4.485
H	2.199	-11.552	-4.970
H	0.818	-11.652	-4.215
O	-1.598	-9.438	-6.959
H	-2.345	-9.546	-6.327
H	-1.961	-8.798	-7.636
O	4.272	-4.141	-6.783
H	5.009	-3.518	-7.046
H	3.484	-3.739	-7.177
H	-1.942	-1.317	-7.941
H	-2.086	-2.361	-6.543
H	-0.970	-4.810	-3.499
H	-0.589	-6.099	-2.358
H	-0.324	1.216	-4.337
H	0.419	1.856	-5.796
H	2.204	1.272	-10.203
H	2.769	0.810	-11.815
H	24.287	-5.148	-5.094
H	22.729	-5.710	-4.578
H	17.647	-7.295	-4.235
H	15.962	-7.779	-4.091
H	8.756	-9.739	-5.527
H	7.308	-10.368	-6.262
H	2.926	-11.295	-7.963
H	1.350	-12.064	-7.867
H	9.104	-8.892	-10.233
H	10.372	-8.089	-9.311
H	11.950	-7.230	-8.673
H	11.717	-5.806	-7.679
H	11.015	-4.816	-6.153
H	11.117	-3.232	-5.361
H	15.417	-0.542	-9.942
H	14.666	-1.657	-11.068
H	13.689	-4.596	-14.151
H	12.757	-5.941	-13.461

211
M467Q FR

C	-1.774	-2.228	-7.679
H	-2.380	-2.930	-8.254
C	-0.279	-2.566	-7.786
H	0.329	-1.862	-7.206
H	0.027	-2.444	-8.830
C	0.020	-3.947	-7.298
N	-0.851	-5.003	-7.514
H	-1.570	-5.023	-8.231
C	-0.387	-6.096	-6.882
H	-0.875	-7.055	-6.898
N	0.734	-5.798	-6.248
C	0.999	-4.461	-6.491
H	1.853	-3.960	-6.064
C	-0.260	-5.424	-3.277
H	-0.083	-6.112	-4.100
C	1.039	-4.684	-2.929
H	0.897	-4.031	-2.061
H	1.284	-4.019	-3.766
C	2.236	-5.562	-2.715
N	3.468	-5.257	-3.279
C	4.317	-6.205	-2.904
H	5.350	-6.267	-3.200
N	3.694	-7.102	-2.120
H	4.070	-7.967	-1.725
C	2.379	-6.719	-1.988
H	1.693	-7.313	-1.408
C	0.392	1.802	-4.618
H	0.177	2.824	-4.303
C	1.785	1.377	-4.091
H	1.729	1.316	-2.998
H	2.519	2.165	-4.305
C	2.332	0.075	-4.599
N	3.038	-0.022	-5.786
H	3.126	0.695	-6.513
C	3.461	-1.291	-5.947
H	4.104	-1.621	-6.752
N	3.062	-2.034	-4.916
C	2.352	-1.192	-4.075
H	1.938	-1.551	-3.145
C	2.667	0.568	-10.657
H	3.691	0.495	-10.290
C	1.908	-0.768	-10.480
H	1.151	-0.898	-11.262
H	1.356	-0.761	-9.530
C	2.821	-1.939	-10.390
N	3.843	-2.196	-11.279
H	4.135	-1.578	-12.040
C	4.548	-3.254	-10.826
H	5.452	-3.610	-11.285
N	4.017	-3.713	-9.697
C	2.942	-2.892	-9.415
H	2.331	-3.060	-8.544
C	23.365	-4.883	-5.141
H	23.188	-3.951	-4.601
C	22.883	-4.760	-6.605
H	23.377	-3.872	-7.016
H	21.812	-4.530	-6.644
C	23.193	-5.955	-7.475
C	22.583	-7.177	-7.389
H	21.798	-7.512	-6.727
N	23.090	-8.040	-8.344
H	22.865	-9.027	-8.337
C	24.086	-7.396	-9.054
C	24.872	-7.824	-10.131
H	24.759	-8.823	-10.547
C	25.767	-6.909	-10.687
H	26.325	-7.176	-11.583
C	25.881	-5.600	-10.172
H	26.570	-4.904	-10.642
C	25.101	-5.179	-9.100
H	25.184	-4.163	-8.724
C	24.178	-6.071	-8.525
C	16.832	-7.314	-4.747
H	16.509	-6.315	-5.032
C	16.958	-8.177	-6.021
H	16.028	-8.045	-6.586
H	17.067	-9.243	-5.782
C	18.073	-7.785	-6.939
N	18.147	-6.518	-7.478
C	19.143	-6.517	-8.333
H	19.448	-5.665	-8.923
N	19.737	-7.745	-8.382
H	20.518	-8.028	-8.955
C	19.077	-8.558	-7.473
H	19.352	-9.593	-7.336
C	7.809	-9.757	-5.382
H	7.564	-10.284	-4.461
C	7.232	-8.332	-5.399
H	7.443	-7.826	-6.348
H	7.757	-7.749	-4.627
C	5.754	-8.303	-5.190
N	5.107	-9.147	-4.301
H	5.540	-9.858	-3.717
C	3.776	-8.989	-4.435
H	3.032	-9.545	-3.888
N	3.531	-8.065	-5.347
C	4.751	-7.630	-5.831
H	4.835	-6.891	-6.610
C	1.987	-11.062	-7.914
H	1.764	-10.520	-6.991
C	1.502	-10.236	-9.126
H	0.407	-10.157	-9.126
H	1.773	-10.723	-10.069
C	2.028	-8.837	-9.064
N	1.178	-7.756	-9.202
H	0.196	-7.821	-9.487
C	1.897	-6.630	-9.007
H	1.506	-5.632	-9.018
N	3.161	-6.920	-8.744
C	3.259	-8.296	-8.781
H	4.196	-8.789	-8.571
C	9.739	-8.166	-10.315
H	10.465	-8.395	-11.099
C	9.091	-6.789	-10.568
H	8.583	-6.847	-11.529
H	9.852	-6.012	-10.690
C	8.102	-6.343	-9.534
N	8.287	-5.222	-8.753
H	9.113	-4.616	-8.747
C	7.206	-5.046	-7.973
H	7.035	-4.177	-7.360
N	6.323	-6.021	-8.179
C	6.879	-6.835	-9.153
H	6.337	-7.694	-9.525
C	12.335	-6.800	-7.913
H	12.221	-7.423	-7.024
C	13.816	-6.520	-8.111
H	14.048	-6.006	-9.039
H	14.304	-7.505	-8.184
S	14.540	-5.603	-6.699
C	10.453	-4.128	-5.778
H	9.850	-3.673	-6.568
C	9.531	-4.680	-4.663
H	10.012	-4.646	-3.677
H	9.325	-5.745	-4.845
C	8.194	-4.026	-4.636
N	7.995	-2.661	-4.575
H	8.734	-1.966	-4.511
C	6.664	-2.419	-4.632
H	6.229	-1.432	-4.634
N	5.990	-3.556	-4.715
C	6.936	-4.562	-4.732
H	6.650	-5.593	-4.870
C	15.618	-1.350	-10.432
H	16.399	-1.164	-11.166
C	15.979	-2.540	-9.525
H	16.049	-3.468	-10.105
H	15.193	-2.745	-8.791
C	17.253	-2.404	-8.759
N	17.637	-3.419	-7.898
C	18.774	-3.046	-7.336
H	19.306	-3.600	-6.577
N	19.141	-1.828	-7.788
H	20.021	-1.373	-7.567
C	18.185	-1.408	-8.695
H	18.260	-0.483	-9.241
C	13.805	-5.466	-13.736
H	14.254	-6.154	-14.463
C	14.675	-5.402	-12.477
H	14.221	-4.800	-11.689
H	15.580	-4.881	-12.776
C	15.088	-6.769	-11.875
H	15.226	-7.512	-12.671
H	14.294	-7.135	-11.217
C	16.357	-6.638	-11.055
O	16.488	-5.846	-10.134
N	17.396	-7.486	-11.408
H	18.302	-7.181	-11.067
H	17.402	-7.869	-12.349
CU	16.504	-4.980	-7.487
CU	4.485	-5.348	-8.736
CU	3.975	-3.717	-4.418
CU	1.875	-7.194	-5.633
O	0.238	-8.663	-4.941
H	0.697	-9.523	-4.797
H	-0.394	-8.854	-5.672
O	6.077	-2.150	-7.600
H	5.715	-2.222	-8.504
H	7.047	-2.119	-7.854
O	1.527	-11.045	-4.481
H	2.214	-11.552	-4.973
H	0.848	-11.684	-4.203
O	-1.602	-9.439	-6.957
H	-2.352	-9.547	-6.329
H	-1.960	-8.799	-7.636
H	-1.940	-1.319	-7.943
H	-2.084	-2.363	-6.544
H	-0.970	-4.811	-3.502
H	-0.591	-6.100	-2.360
H	-0.307	1.201	-4.334
H	0.432	1.843	-5.796
H	2.206	1.277	-10.204
H	2.771	0.813	-11.816
H	24.287	-5.147	-5.094
H	22.728	-5.710	-4.579
H	17.646	-7.295	-4.234
H	15.961	-7.778	-4.090
H	8.756	-9.742	-5.526
H	7.309	-10.371	-6.263
H	2.926	-11.293	-7.960
H	1.350	-12.061	-7.868
H	9.082	-8.869	-10.229
H	10.352	-8.067	-9.310
H	11.943	-7.229	-8.677
H	11.711	-5.808	-7.678
H	11.011	-4.821	-6.146
H	11.116	-3.234	-5.356
H	15.417	-0.542	-9.942
H	14.667	-1.657	-11.067
H	13.688	-4.596	-14.151
H	12.757	-5.940	-13.460
